# In situ observation of the percolation threshold in multiphase magma analogues

**DOI:** 10.1007/s00445-020-1370-1

**Published:** 2020-03-04

**Authors:** M. Colombier, F. B. Wadsworth, B. Scheu, J. Vasseur, K. J. Dobson, F. Cáceres, A. Allabar, F. Marone, C. M. Schlepütz, D. B. Dingwell

**Affiliations:** 1 Earth and Environmental Sciences, Ludwig-Maximilians-Universität, Theresienstr. 41, 80333 Munich, Germany; 2 Department of Earth Sciences, Durham University, Durham, DH1 3LE UK; 3 Centre for Advanced Study, Ludwig-Maximilians-Universität, Munich, Germany; 4 Department of Geosciences, University of Tübingen, Tübingen, Germany; 5 Swiss Light Source, Paul Scherer Institute, 5232 Villigen PSI, Switzerland

**Keywords:** Effusive-explosive transition, Percolation threshold, Outgassing, Crystal-rich magma, Magma viscosity, Gas overpressure, Porosity, Pore connectivity, Hysteresis, Strombolian/Vulcanian eruptions, Dome-forming eruptions

## Abstract

Magmas vesiculate during ascent, producing complex interconnected pore networks, which can act as outgassing pathways and then deflate or compact to volcanic plugs. Similarly, in-conduit fragmentation events during dome-forming eruptions create open systems transiently, before welding causes pore sealing. The percolation threshold is the first-order transition between closed- and open-system degassing dynamics. Here, we use time-resolved, synchrotron-source X-ray tomography to image synthetic magmas that go through cycles of opening and closing, to constrain the percolation threshold *Φ*_*C*_ at a range of melt crystallinity, viscosity and overpressure pertinent to shallow magma ascent. During vesiculation, we observed different percolative regimes for the same initial bulk crystallinity depending on melt viscosity and gas overpressure. At high viscosity (> 10^6^ Pa s) and high overpressure (~ 1–4 MPa), we found that a *brittle-viscous* regime dominates in which brittle rupture allows system-spanning coalescence at a low percolation threshold (*Φ*_*C*_~0.17) via the formation of fracture-like bubble chains. Percolation was followed by outgassing and bubble collapse causing densification and isolation of the bubble network, resulting in a hysteresis in the evolution of connectivity with porosity. At low melt viscosity and overpressure, we observed a viscous regime with much higher percolation threshold (*Φ*_*C*_ > 0.37) due to spherical bubble growth and lower degree of crystal connection. Finally, our results also show that sintering of crystal-free and crystal-bearing magma analogues is characterised by low percolation thresholds (*Φ*_*C*_ = 0.04 – 0.10). We conclude that the presence of crystals lowers the percolation threshold during vesiculation and may promote outgassing in shallow, crystal-rich magma at initial stages of Vulcanian and Strombolian eruptions.

**Electronic supplementary material:**

The online version of this article (10.1007/s00445-020-1370-1) contains supplementary material, which is available to authorized users.

## Introduction

Permeability controls the efficiency with which exsolved volatiles can escape from the magma and are released to the atmosphere or conduit walls before and during volcanic eruptions (Jaupart and Allègre 1991). The onset, longevity and magnitude of the magma permeability all affect the rates of gas overpressure increase or reduction, which may control eruption explosivity. Magma permeability evolution in a volcanic conduit is affected by processes such as bubble coalescence (Eichelberger et al. 1986; Lindoo et al. 2017), brittle fracturing (Tuffen and Dingwell 2005; Kushnir et al. 2017; Lamur et al. 2017), compaction (Westrich and Eichelberger 1994; Michaut et al. 2009; Heap et al. 2015; Gonnermann et al. 2017) or granular densification in particle-filled fractures and veins (Okumura and Sasaki 2014; Kendrick et al. 2016). This evolution of magma permeability can show a hysteresis with vesiculation followed by outgassing and compaction or fragmentation followed by welding (e.g. Rust and Cashman 2004; Wright et al. 2009; Michaut et al. 2009; Okumura et al. 2013). The percolation threshold *Φ*_*C*_ is defined as the critical porosity at which the transition from impermeable to permeable magma occurs and therefore represents the divide between a chemically and physically closed system and one that is open.

Previous work has shown that *Φ*_*C*_ can vary in magmas significantly (Blower 2001; Rust and Cashman 2004; Burgisser et al. 2017; Colombier et al. 2017; Lindoo et al. 2017; Gonnermann et al. 2017; Giachetti et al. 2019). This variation depends on the process that is operative in effecting the porosity change (e.g. is it vesiculation, fracture propagation or welding? Colombier et al. 2017), the pore size distribution (Blower 2001; Burgisser et al. 2017), the degree of shear deformation and fracturing during flow (Kushnir et al. 2017) and groundmass crystallinity (Lindoo et al. 2017; deGraffenried et al. 2019) among many other factors.

The effect of crystals on vesiculation has been examined theoretically (Blower 2001) and experimentally (e.g. Bai et al. 2011; Okumura et al. 2012; Oppenheimer et al. 2015; Pistone et al. 2015a; Parmigiani et al. 2016; Spina et al. 2016; Lindoo et al. 2016, 2017; deGraffenried et al. 2019). Most of these studies showed that the presence of crystals can lead to a reduction of *Φ*_*C*_ and an increase of permeability due to (i) increased melt porosity and pore connectivity at a given bulk porosity (Blower 2001) and (ii) enhanced bubble deformation and migration via the formation of fingering and fracture-like geometries during coalescence (e.g. Oppenheimer et al. 2015; Parmigiani et al. 2016; Lindoo et al. 2017). Although these studies provided insightful results on the influence of crystals on *Φ*_*C*_, a four-dimensional quantitative analysis of percolation in magma analogues with crystallinities, viscosities and gas pressures relevant to Vulcanian and Strombolian conditions is still lacking.

In situ 4D synchrotron X-ray computed tomography can shed light on the real-time evolution of pore characteristics such as porosity, pore connectivity, permeability and pore size distribution during vesiculation (Baker et al. 2012; Pistone et al. 2015b) and densification (Wadsworth et al. 2017). Here, we perform in situ vesiculation and densification experiments on a range of crystal-bearing samples at a synchrotron X-ray computed tomography beamline and track the evolution of the pore connectivity with porosity during the experiments. We quantify the percolation thresholds during vesiculation, outgassing and sintering and use the results to provide quantitative constraints that can be used to update models of magma ascent that incorporate dynamic permeability changes and the transition from closed- to open-system degassing dynamics.

## Materials and methods

### Synthesis of the magma analogues

All experiments were performed at the TOMCAT facility, a beamline for tomographic microscopy and coherent radiology experiments (Stampanoni et al. 2006) located in the Swiss Light Source synchrotron radiation source at the Paul Scherer Institute in Switzerland.

We synthesised bubble-bearing, gas-overpressured, and moderately to highly crystalline magma analogues by sintering soda-lime-silica glass beads (Potters Industries; initial particle size, 63–90 μm in diameter; composition given in Wadsworth et al. 2014, Table 1) with controlled proportions of solid crystals (quartz) of the same size (0–30 vol.%). We first packed the granular mixtures of glass beads and crystals into cylindrical samples of approximately 2.5 mm height and 2.5 mm diameter and sintered them at 850 °C and 5 MPa applied using argon gas as the pressurising medium in a pressure autoclave. The samples were heated at 15 K min^−1^ and held at 850 °C for 8 h, sufficient for complete sintering (see Wadsworth et al. (2016) for predictions of sintering dynamics) to dense magma analogues with low porosities (*Φ* = 0.04) and isolated, impermeable bubbles containing argon at 5 MPa overpressure relative to ambient quench conditions. The samples were then cooled in isochoric conditions before decompression. During cooling, the gas pressure in the gas pocket above the sample drops similarly to the gas pressure in the bubbles, maintaining equilibrium and the bubbles do not shrink significantly. This also means that as the sample melt passes through the glass transition interval (*T*_g_), the gas pressure in the bubbles drops to 3.7 MPa down to 1.3 MPa at room temperature. However, on reheating, this is reversible such that the sample crosses *T*_g_ again and the gas pressure is returned to 3.7 MPa.

**Fig. 1:**
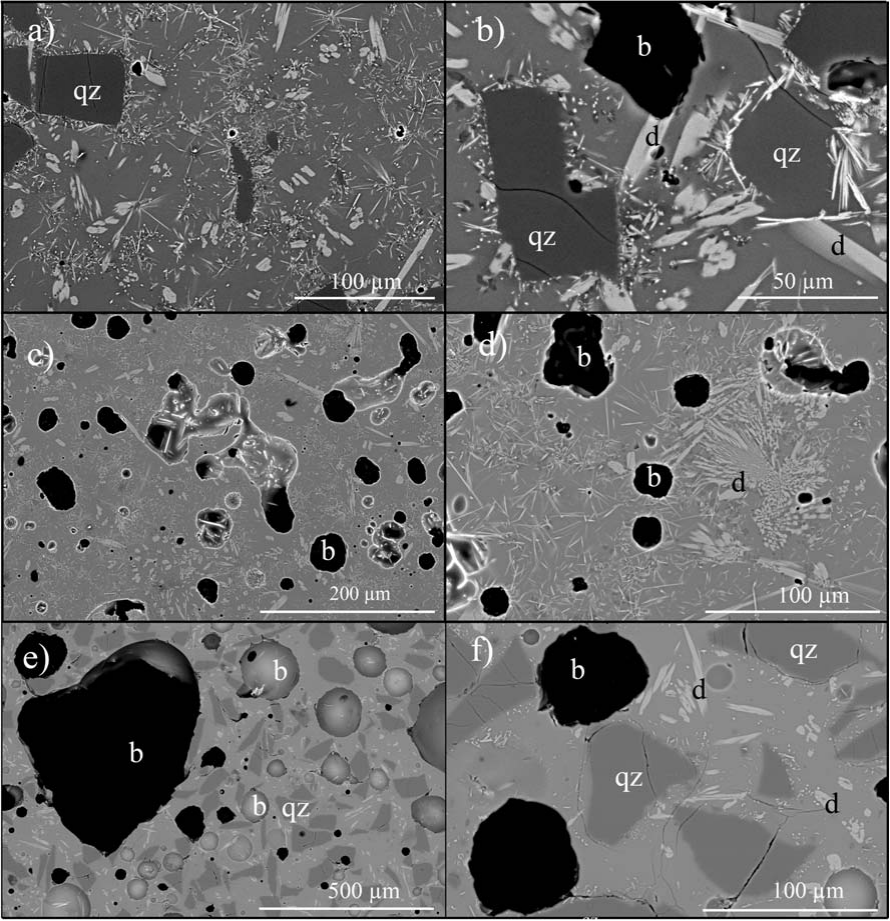
SEM backscattered images showing the pre-experimental textures after synthesis (**a**, **b**) and the post-experimental textures after the vesiculation experiments (**c**–**f**). Some quartz crystals with a grey to dark grey colour are marked ‘qz’. Devitrite microlites Na_2_Ca_3_Si_6_O_16_ formed during synthesis appear in a pale grey colour and some are marked ‘d’. Vesicles have a grey to black colour and some of them are marked ‘b’. **a** Sample DRY1-25-pre-after synthesis showing the presence of quartz crystals and abundant needle-shaped devitrite microlites, with a total crystallinity of 25 vol.%. **b** Zoom of (**a**) showing rims of devitrite microlites around quartz crystals and few isolated vesicles. Note the high local crystallinity in this sample. **c** Sample DRY1-14-3 after a vesiculation experiment showing a network of isolated bubbles in a matrix of glass and devitrite microlites. This sample has a total crystallinity of 14 vol.%. **d** Zoom of (**c**) showing clusters of devitrite microlites causing high local crystallinity. **e** Sample DRY2-44-4 after a vesiculation experiment showing large spherical to sub-spherical isolated vesicles in a groundmass of quartz and devitrite crystals. The total crystallinity of this sample is 44 vol.%. **f** Zoom of (**e**) illustrating the heterogeneous crystallinity

The magma analogues contain a population of pre-mixed quartz crystals (0, 10 and 30 vol.%) and devitrite Na_2_Ca_3_Si_6_O_16_ (stoichiometry after Knowles and Thompson 2014) or additional minor phases that grew during synthesis (Fig. 1), yielding final bulk crystallinities of 14–48 vol.% (see Table DR1). Devitrite has a composition similar to that of the glass, which means that the glass composition did not evolve significantly during crystallisation. The cause of the crystallisation is unknown but likely relates to the high-temperature steel autoclave or the high gas pressures (5 MPa) used here, because glass beads of the same composition sintered at similar Ar-gas temperature conditions did not crystallise (see Wadsworth et al. 2014, 2016). Heterogeneous distribution of crystals leads to local crystallinities higher than 45 vol.% even in the samples with low bulk crystallinity (Fig. 1). The isolated bubbles are mostly localised in these crystal-rich areas (Fig. 1). In most of the samples, we did not observe any notable modifications of the crystal networks before and after the in situ vesiculation experiments. However, the degree of crystal connectivity seems to decrease locally in the post-experimental products (e.g. Fig. 1f).

We chose a combination of soda-lime glass beads and Argon gas in order to study vesiculation process for several reasons. First, the samples were nominally water-free, meaning that when they were re-heated after synthesis, any bubble growth or bubble size changes are related only to bubble expansion by gas volume and pressure variations, rather than by diffusion of volatiles (Ar solubility in the melt is low < 0.06 wt%; Caroll and Stolper 1991). Therefore, our experiments represent a necessary simplification of natural processes, allowing us to isolate *Φ*_*C*_ as a function of crystallinity only. Second, soda-lime-glass beads have been widely used for sintering experiments, including previous work at the TOMCAT (Wadsworth et al. 2017). Thus, choosing soda-lime magma analogue allowed us to compare vesiculation and sintering experiments on the same material, to compare the porosity-connectivity trends from this study to previous work and also to calibrate the temperature of the laser heating system at the beamline (see the Electronic supplementary material).

### Vesiculation and sintering experiments at the synchrotron

At the synchrotron, we performed two suites of X-ray tomography experiments using a GigaFRoST camera (Mokso et al. 2017) which has a pixel size of 11 μm and a chip size of 2016 × 2016 pixels. These specificities combined with the optical magnification of the microscope of × 6.8 yield an effective pixel size for the measurements of 1.6 μm. For the first set of experiments, the magma analogues obtained after synthesis were loaded into lidded alumina sleeves and were heated at ambient pressure above the glass transition interval using the in situ laser heating system at the beamline (Fife et al. 2012). The alumina sleeves were large enough (3.0 mm inner diameter) so that the melts were not exposed to confinement at the time the data shown here were collected. Temperatures of the alumina sleeve were calibrated by sintering standard glass powders and comparison with the ex situ results of Wadsworth et al. (2016; see the Electronic supplementary material), resulting in temperatures within 10 °C of those measured by pyrometry (Fife et al. 2012). The samples were heated to 550–930 °C, resulting in melts (excluding crystals) with viscosities ranging from 10^12^ to 10^3^ Pa s (using the viscosity model in Wadsworth et al. 2014 for the same composition of glass beads), at heating rates of 10–80 K min^−1^. All samples were heated linearly without isothermal dwells. This means that a single sample tracks through the regimes that we identify. In essence, this means that each sample undergoes its own hysteresis pathway of closed-to-open-system dynamics, similar to the way natural magmas might evolve, albeit in a simplified laboratory setting.

Although we could not directly measure the internal pore pressure during our experiments, we could estimate its evolution after synthesis and during heating-induced vesiculation at the synchrotron using equation of state with an initial gas pressure of 5 MPa at 850 °C in the autoclave. In our experiments, the porosity and total volume of cut-out ‘regions-of-interest’ in our samples are measured variables, the product of which is the gas volume or pore volume (*V*_g_), which is a function of time during heating. As stated in the ‘Materials and methods’, the bubble gas pressure at *T*_g_ is *P*_i_ = 3.7 MPa, which we take to be a starting condition. At temperatures above *T*_g_, the gas pressure (or pore pressure) *P*_p_, is then *P*_p_ = *P*_i_*V*_g, i_*T*/(*T*_g_*V*_g_), where *P*_i_ is the pressure of the bubbles at *T*_g_ (which is 3.7 MPa, as discussed), *V*_g, i_ is the initial volume of the gas (or pores) at *T*_g_, and *T* is the evolving temperature. Because we take the measured porosity of the samples as an input to this calculation of *P*_p_, this is not affected by whether or not the bubbles are at equilibrium volume. We assumed that during percolation, all the gas escaped to the exterior of the sample causing an instantaneous pressure drop to the atmospheric pressure (Westrich and Eichelberger 1994). For the second set of experiments, we performed sintering experiments in situ using glass beads or glass beads mixed with 40 vol.% quartz crystals using the in situ method described in Wadsworth et al. (2017).

### Porosity and pore connectivity measurements

Each 3D scan was acquired in 1 s with a 5-s wait time between scans and the 3D volumes were visualised and quantified using Avizo (https://www.thermofisher.com/de/de/home/industrial/electron-microscopy/electron-microscopy-instruments-workflow-solutions/3d-visualization-analysis-software.html). A detailed description of the scanning procedure and the image processing strategy used to segment the pore networks in each image is given in the Electronic supplementary material. Porosity was defined as the total volume of pores divided by the volume of interest analysed. Pore connectivity was measured in two distinct ways using the X-ray images. First, we measured a percolative connectivity *C*, defined as the fraction of volume of pores that could be traced continuously from face-to-face in the volume of interest, relative to the total volume of pore space. Such percolative connectivity is relevant because it allows to distinguish permeable (*C* > 0) and impermeable (*C* = 0) porous networks, allowing comparison with permeability data. *C* was measured in three mutually orthogonal directions and the maximum value was taken. Second, a pycnometer-like definition of connectivity *C*′ was quantified, in which all the pores connected to the exterior of the samples are also counted as connected, although they do not necessarily contribute to permeability. We quantified *C*′ in order to compare our experimental data with natural data on volcanic rocks obtained by He-pycnometer. For both definitions, connectivity was obtained by dividing the connected porosity by the total porosity. The reader is referred to Colombier et al. (2017, 2018) for a discussion of the different connectivity definitions. We also discuss the uncertainties caused by image processing on the percolation threshold and provide a way to convert connectivity data from one method/definition to the other in the Electronic supplementary material.

## Results

The experimental conditions and raw data are given for each vesiculation and sintering dataset in Table DR1 in the Electronic supplementary material.

### Vesiculation experiments

**Fig. 2:**
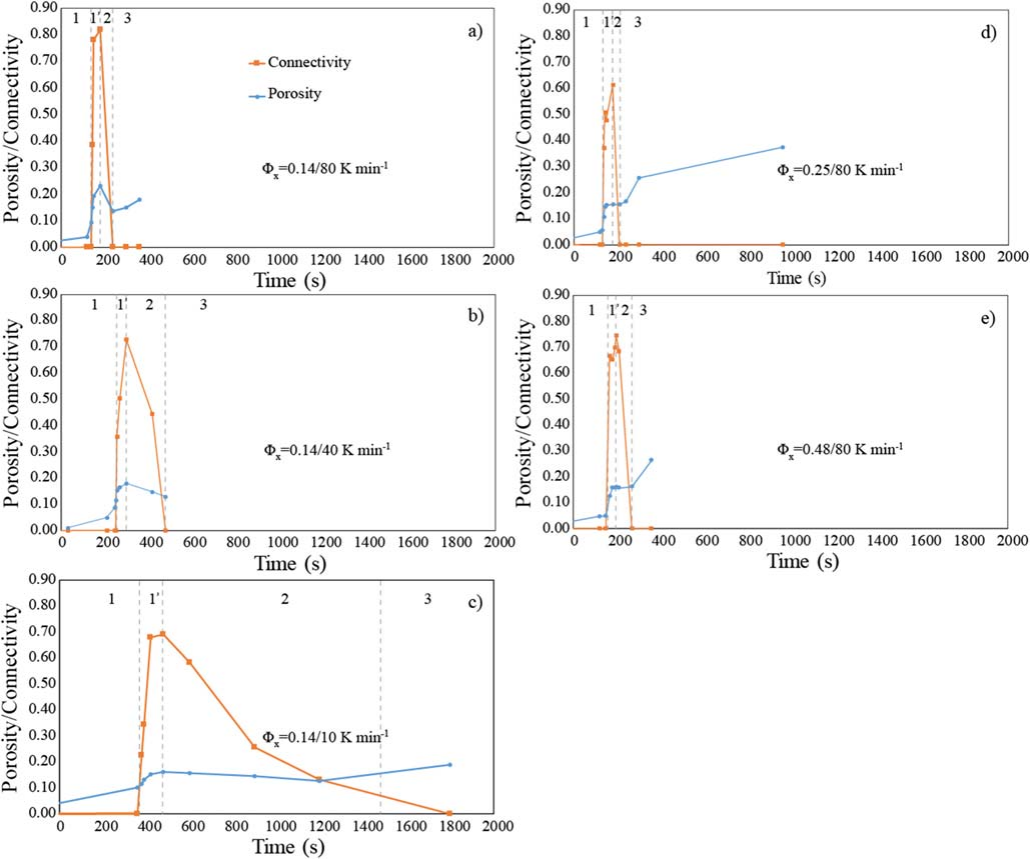
Evolution of porosity and percolative connectivity with time during the single experiments. **a**–**c** Sample DRY1-14-34, DRY1-14-42 and DRY1-14-31 with bulk crystallinity of 14 vol.% and heating rates of 80, 40, and 10 K min^−1^, respectively. **d** Sample DRY2-25-38 with a bulk crystallinity of 25 vol.% at a heating rate of 80 K min^−1^. **e** Sample DRY2-48-46 with a bulk crystallinity of 48 vol.% at a heating rate of 80 K min^−1^. The different regimes of sample response are illustrated. Regime 1 corresponds to the *brittle-viscous* regime with 1′ indicating the onset and increase of connectivity at *Φ*_*C*_. Regime 2 corresponds to the *outgassing* regime with reduction of connectivity until complete isolation (*C* = 0). Regime 3 corresponds to the *viscous* regime with increase of porosity and no percolation (*C* = 0). Errors on porosity and connectivity are smaller than symbols

**Fig. 3:**
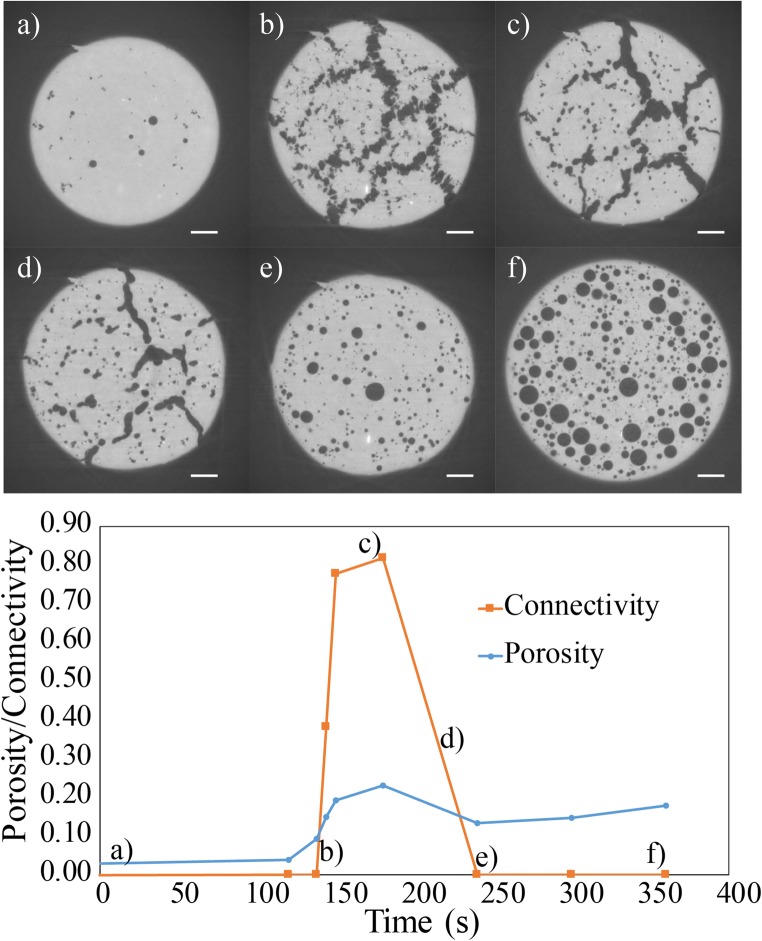
Evolution of pore geometry during a single vesiculation experiment in the sample DRY1-14-34 at different regimes of sample response. **a** Growth of isolated bubbles in the *brittle-viscous* regime. **b** Onset of connectivity and formation of fracture-like chains of coalescing bubbles in the brittle-viscous regime. **c** System-spanning connectivity caused by complete coalescence and formation of smooth, permeable pathways allowing gas escape to the exterior of the sample marking the transition from brittle-viscous to *outgassing* regime. **d** Progressive densification and isolation of the porous network with reduction of connectivity caused by pore collapse in the outgassing regime. **e** Complete isolation of the porous network following the outgassing regime resulting in a polydisperse bubble size distribution with spherical bubbles. **f** Growth of the spherical, isolated bubbles causing expansion of the system and increase of porosity with no percolation in the *viscous* regime. The scale bars represent 500 μm. The evolution of porosity and connectivity for this sample with time is illustrated in the bottom panel

We identified three main successive regimes of sample response during heating in the vesiculation experiments. These regimes can be easily distinguished for each experiment by tracking the evolution of porosity and percolative connectivity, *Φ* and *C*, as a function of time (Fig. 2) as follows. First, we see initial bubble growth with only localised coalescence leading to a slight increase of porosity with no onset of connectivity (*C* = 0). This step is followed by percolation via system-spanning connecting bubble chains with a mixed fracture and bubble-like microstructural geometry causing steeper increase of porosity and onset of connectivity (*C* > 0) up to a maximum value (*C*_max_ = 0.69–0.82). Second, gas escape through the interconnected bubble chains occurs causing densification and bubble collapse with a dramatic reduction of connectivity up to *C* = 0 (isolation) and a minor drop of porosity. Finally, we see a second stage of bubble growth in which the bubbles grow spherically and remain isolated, pushing the crystal-bearing melt aside with a marked increase of porosity but no percolation (*C* = 0). For convenience, we name these qualitative regimes according to our interpretation of the dominant physical processes involved: (1) *brittle-viscous*, (2) *outgassing* and (3) *viscous* regimes*.* Typical 2D textures corresponding to these different regimes are illustrated for sample DRY1-14-34 in Fig. 3. Although the bubble number density seems to increase in the viscous regime (Fig. 3e, f), we stress that it remains in fact constant as there is neither bubble nucleation due to the absence of volatiles nor bubble coalescence occurring in this regime. This seemingly increasing number density is simply related to the smaller size of bubbles in the early stage of bubble growth (Fig. 3e).

**Fig. 4:**
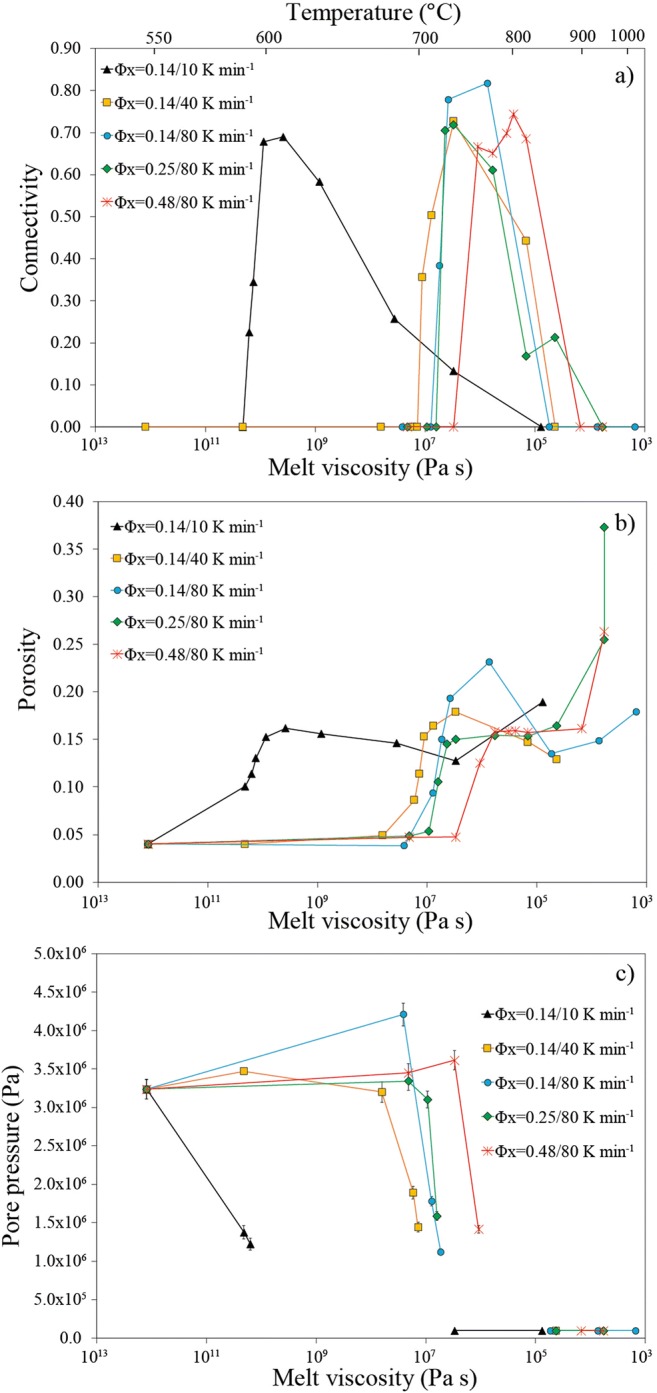
Evolution of **a** percolative connectivity, **b** porosity and **c** pore pressure as functions of melt viscosity for the different vesiculation experiments. In (**c**), the pore pressure was assumed to drop instantaneously to the atmospheric pressure at the time of percolation, explaining the gap in data during percolation and outgassing. The maximum error on viscosity is 1 log unit (see Table DR1 in the Electronic supplementary material)

We note that the melt temperature (*T*) and melt viscosity (*η*) increases and decreases, respectively, from the regimes (1) to (3) during continuous heating. To illustrate this, we plot the evolution of *Φ* and *C* for each experiment as a function of *T* and *η* (Fig. 4). We first observe that the brittle-viscous regime with onset and increase of connectivity and fracture-like geometries occurred at 10^5^ < *η* < 10^10^ Pa s, whereas the viscous regime with spherical bubble growth occurred mostly at 10^3^ < *η* < 10^5^ Pa s. Viscosities in the outgassing regime are intermediate. We also see in Fig. 4 that the brittle-viscous and outgassing regimes occur at much higher viscosity in the single experiment at low heating rate (10 K min^−1^) than in the ones at high heating rates (40–80 K min^−1^).

**Fig. 5:**
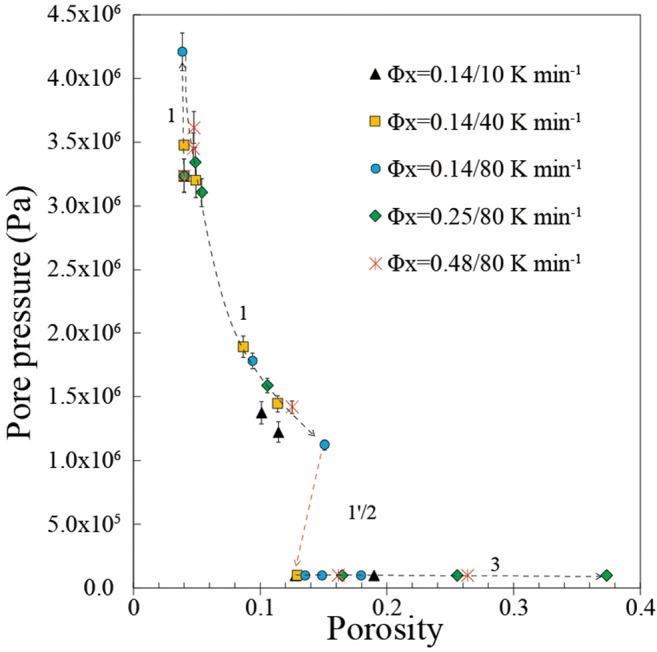
Evolution of pore pressure with porosity in the vesiculation experiments. Pore pressure is calculated assuming that the measured total gas volume in the sample follows its equation of state as the temperature increases (see text). The experiments at 40 and 80 K min^−1^ fall on a similar trend suggesting initial increase of pressure at constant initial porosity (*Φ* = 0.04) due to heating, followed by a decrease of pore pressure related to the increase of porosity. During percolation and subsequent gas escape, the pore pressure decreases to atmospheric pressure (arrow 1′/2 with 1′ referring to the onset and increase of connectivity in the *brittle-viscous* regime and 2 corresponding to the subsequent *outgassing* regime; see Fig. 2). Finally, the pore pressure remains constant at atmospheric pressure in the *viscous* regime. The single experiment at 10 K min^−1^ follows a subtly different trend at lower pore pressure before percolation. This is likely because there was no initial increase of pore pressure at the starting porosity because vesiculation occurred at lower temperature for such low heating rate. The black dashed curves are constrained from datapoints in the brittle-viscous and viscous regimes, whereas there is a gap of data in the outgassing regime, and the red arrow is to guide the eye towards the overall reduction of porosity and pore pressure during outgassing

Finally, we estimated the evolution of gas overpressure during the vesiculation experiments using equation of state (Figs. 4c and 5). We observe that vesiculation in the brittle-viscous regime occurred in the presence of large initial bubble gas overpressure (~ 10^6^ Pa resulting from synthesis) whereas the argon overpressure was lost during percolation and outgassing (Fig. 4c). The gas pressure therefore dropped to atmospheric pressure and remained constant during subsequent vesiculation in the viscous regime in which growth of bubbles was due to temperature increase only by equation of state (Fig. 4c). In the brittle-viscous regime, we also note that the internal gas pressure is on average lower at low heating rates (10 K min^−1^) than at high heating rates (40 to 80 K min^−1^; Figs. 4c and 5).

**Fig. 6:**
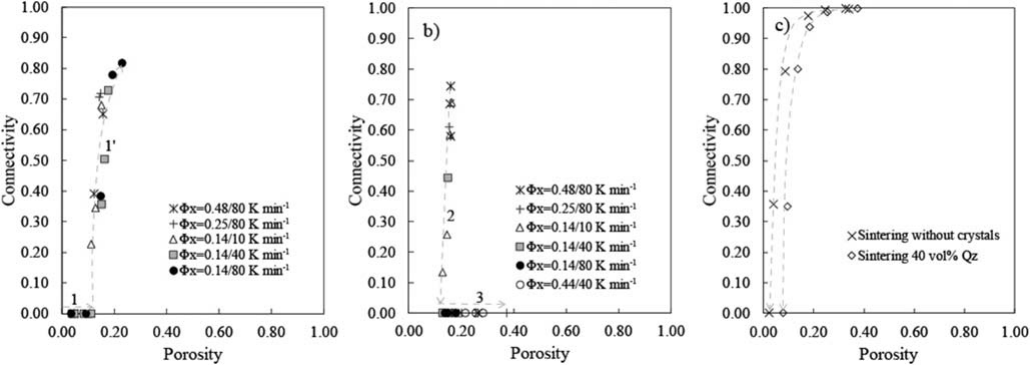
Percolative connectivity (*C*) evolution with porosity (*Φ*) relationships in the vesiculation and sintering experiments. **a***C* vs. *Φ* trend in the *brittle-viscous* regime with onset of connectivity at a *Φ*_*C*_ = 0.17. The arrow 1 corresponds to bubble growth and porosity increase without percolation at *Φ*< *Φ*_*C*_ whereas 1′ indicates the onset and increase of connectivity at *Φ*_*C*_. **b***C* vs. *Φ* trend during the *outgassing* regime (densification) and further bubble growth in the *viscous* regime. **c***C* vs. *Φ* trends of the sintering experiments with 40 vol.% crystals (open diamonds) and without crystals (black crosses). The arrows in each plot serve to guide eye towards the direction of the *C*–*Φ* paths

When percolative connectivity of the pore network across the sample, *C*, is plotted as a function of porosity, *Φ* (Fig. 6a, b), we can identify the percolation thresholds for each regime. The percolation threshold in the brittle-viscous regime was marked by an abrupt increase of *C* from *C* = 0 as *Φ* increases during the experiments (Fig. 6a) and occurred systematically at *Φ*_*C*1_~0.17 ± 0.06, independent of the sample total crystallinity, melt viscosity, heating rate or overpressure at the time of percolation. Once these pore pathways connected to the exterior of the sample, the outgassing regime occurred leading to subsequent pore collapse, densification and isolation of the connected porosity leading to a hysteresis in the *C* vs. *Φ* path towards a new percolation threshold 0.13 < *Φ*_*C*2_ < 0.16 (see Fig. 6b). After complete isolation, further temperature increase led to expansion of the bubbles in the viscous regime up to *Φ* = 0.37 (Fig. 6b) without percolation.

### Sintering experiments

**Fig. 7:**
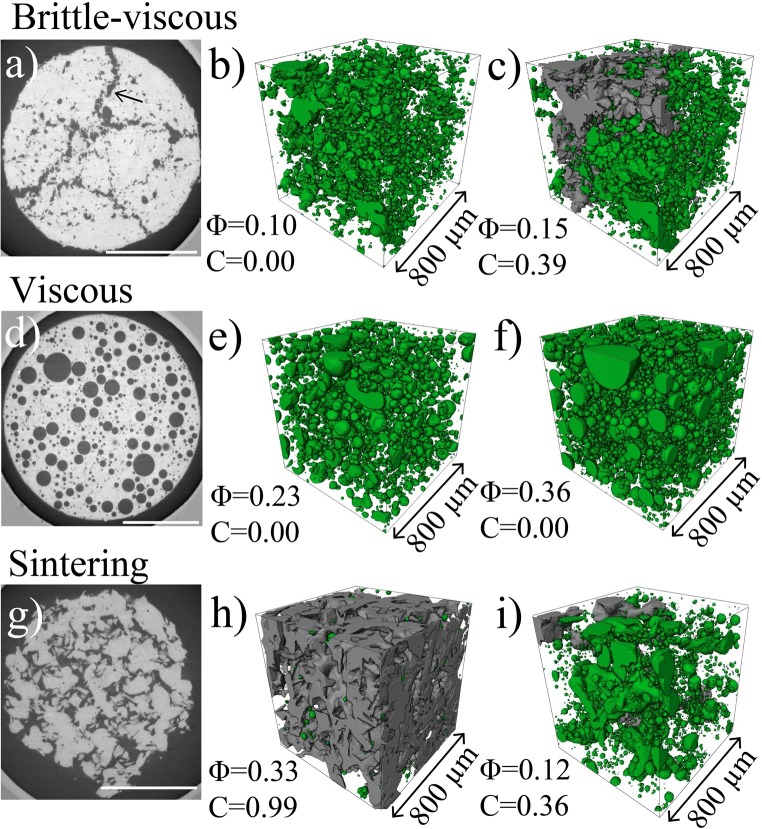
X-ray tomography textural images of crystal-rich synthetic magmas used here. **a**–**c** 2D tomography image (**a**) and 3D volume renderings (**b**, **c**) showing the bubble-chain pathways and the evolution of connectivity with porosity in the *brittle-viscous* regime. **d**–**f**) 2D tomography image (**d**) and 3D volume renderings (**e**–**f**) showing the increase of porosity without onset of connectivity in the *viscous* regime. **g**–**i** 2D tomography image (**g**) and 3D volume renderings (**h**, **i**) showing the evolution of connectivity with porosity in the *sintering* regime with 40 vol.% quartz crystals. Connected and isolated bubbles/pores are represented in grey and green in the volume renderings, respectively. Scale bars in (**a**), (**d**) and (**g**) correspond to 1 mm

In the *sintering* regime, the intergranular porous network was initially fully connected and progressively densified and became isolated (Fig. 6c). During sintering without crystals, *C* decreased dramatically with *Φ* towards a very low percolation threshold *Φ*_*C*3_~0.04 (Fig. 6c). Sintering experiments with 40 vol.% quartz crystals show a similar trend but at higher *Φ*, yielding a higher percolation threshold *Φ*_*C*3_~0.10 (Fig. 6c). Figure 7 shows the typical 2D and 3D textures and evolution of connectivity observed in the brittle-viscous, viscous and sintering regimes.

## Discussion

### Regimes for magma outgassing during vesiculation

Our results allow us to separate vesiculation processes into those which resemble classic bubble growth (the viscous regime described here; Proussevitch et al. 1993; Lindoo et al. 2017), and those which resemble fracture-driven processes (the brittle-viscous regime described here). Our results unify those regimes under the conceptual framework in which the former is typical of low melt viscosity (< 10^6^ Pa s), or low gas overpressure systems (0.1 MPa), while the latter is typical of high viscosity (> 10^6^ Pa s), or high gas overpressure systems (1–4 MPa).

### Brittle-viscous regime

In the brittle-viscous regime, system-spanning coalescence and percolation occur at *Φ*_*C*1_~0.17, which is much lower than in the viscous regime (Fig. 6) for similar bulk crystallinities. We note that the bubbles start to grow, and then rapidly interconnect via elongate pore structures that resemble blunted fractures (Figs. 3b–d and 7). We hereafter propose that this peculiar vesiculation process is related to the high viscosity (> 10^6^ Pa s) and high overpressure (1–4 MPa) in the brittle-viscous regime relative to the viscous regime.

We first note that the formation of percolating, fracture-like bubble chains occur in areas of the sample that are locally concentrated in crystals such that the local crystallinity where these features form is always *ϕ*_*x*_ ≥ 0.45. The increase of the bulk suspension viscosity around pores in locally crystal-rich areas (Mueller et al. 2011a) and the high melt viscosity (> 10^6^ Pa s) at the relatively low temperatures used (compared with the viscous regime) both mean that the resistance to expansion of the over-pressured bubbles is high. These observations lead us to conclude that local brittle behaviour dominates. The timescale for purely viscous dissipation of the overpressure Δ*P* = 1 − 4 MPa (Fig. 5) by bubble expansion is *λ* = *η*_a_/Δ*P* where *η*_a_ is the viscosity of the crystal-melt mixture. This is then maximally 10^2^ < *λ* < 10^8^ s for ${\phi}_x^{\prime }=0.45$ using the model for *η* from Mueller et al. (2011a) with the estimated maximum packing of crystals in a melt *ϕ*_*m*_ = 0.5 typical of crystals with moderate to high aspect ratios (Mueller et al. 2011a) and a melt viscosity 10^6^ < *η* < 10^12^ Pa s. The equant quartz crystals likely had a limited influence on bubble coalescence; hence, we chose a maximum packing relevant for the elongated devitrite crystals. Here, we use ${\phi}_x^{\prime }$, rather than *ϕ*_*x*_, where the prime denotes the value local to the bubble walls. We note that this local value of ${\phi}_x^{\prime }>0.45$ was the case only for vesiculation in the brittle-viscous regime, whereas for the viscous regime, the system expanded as a continuum and therefore *ϕ*_*x*_ is appropriate. The time at which *C* increases due to the formation of apparent fracture-like pores is much less than *λ*, confirming that for this regime there was insufficient time for purely viscous processes to complete.

As the experiment progresses and the temperature increases, it is clear that the gas overpressure, which grows due to the equation of state of the gas phase in the isolated pores, cannot be dissipated and instead induces shear stresses in the melt. We suggest that these were sufficient to rupture the walls of the growing bubbles and interconnect them at lower bulk *Φ* than would have occurred without brittleness (see Forte and Castro 2019 for a similar process in natural samples).

The influence of heating rate on the onset of connectivity and percolation (Fig. 4a) is likely related to the competing effect of melt viscosity and gas overpressure while the temperature increases in the brittle-viscous regime. At low heating rate (10 K min^−1^), percolation occurs at higher viscosity (10^10^ Pa s) which promotes a brittle behaviour, but the gas overpressure is lower (1 MPa; Figs. 4a, c; 5). At high heating rates (40–80 K min^−1^), the viscosity at time of percolation is lower (10^6^–10^7^ Pa s) but the average gas pressure is higher (3–4 MPa) likely promoting rupture and bubble coalescence (Figs. 4c and 5). The higher gas pressure for higher heating rates can be explained by the fact that temperature increased significantly before the increase in porosity, leading to an initial increase of pore pressure (Fig. 4c). At low heating rate (10 K min^−1^), the pressure rise due to heating was negligible compared with pressure reduction due to the increase in gas volume fraction (porosity) because of the lower temperature, explaining the absence of initial pressure increase and the lower average pressure for low heating rate in the brittle-viscous regime (Figs. 4c and 5). Despite of these differences induced by the range of heating rates of our experiments, the key conclusion here is that a combination of higher viscosity and gas overpressure in the brittle-viscous regime relative to the viscous regime can explain brittle rupture and the fracture-like geometries developed during coalescence.

Texturally similar fracture-like percolation has also been explained by gas-filter pressing. Oppenheimer et al. (2015) found low percolation thresholds, similar to our brittle-viscous regime. However, their experimental system was not percolating due to local brittleness, and rather the gas phase was filter pressing between the crystals, expelling the liquid. They also observed that as *ϕ*_*x*_ → *ϕ*_*m*_, the geometry of the advancing gas phase approached a fracture-like form. We found no direct evidence for such gas-filter pressing in our experiments. This suggests that similar percolative responses at low percolation thresholds can be achieved by different processes when the pores localise on fracture-like geometries.

### Outgassing regime

Gas escape occurred instantaneously when the fracture-like bubble chains connected to the exterior of the sample. Such system-spanning connection of the bubble pathways causes a rapid reduction of the pore pressure towards atmospheric pressure (Westrich and Eichelberger 1994), which is rate-limited by the evolving permeability of the sample. When the samples reached a maximum connectivity, the permeability was high and gas pressure equilibration is likely to have been rapid. Once the gas and melt pressures equilibrate, the excess surface pressure (arising from the surface tension and the local curvature of the now-interconnected pore space) acts to collapse the bubbles again (Kennedy et al. 2016). This leads to a reduction of porosity, pore connectivity and pore aperture size. Therefore, we also expect important decrease of permeability during this densification and isolation of the system. We note that the succession of vesiculation and outgassing produces a hysteresis in the *C* – *Φ* path as observed in previous experiments of vesiculation followed by compaction (Okumura et al. 2013). We expect a similar hysteresis for the evolution of permeability with porosity, as proposed by Rust and Cashman (2004). A main difference in the hysteresis observed in our experiment and previous studies is that the percolation threshold during initial vesiculation is lower than the one during subsequent outgassing and pore collapse. This peculiar hysteresis may be due to the decrease in viscosity during outgassing, which likely caused a progressive transition towards the viscous regime and a shift of the percolation threshold towards higher values (0.13 < *Φ*_*C*2_ < 0.16).

### Viscous regime

The percolation threshold was not achieved in our experiments in the viscous regime and must therefore be significantly higher than in the brittle-viscous regime (*Φ*_*C*_ > 0.37) for a similar initial bulk crystallinity. In this regime, the lower bulk viscosity allows the bubbles to expand spherically while pushing the crystals apart. The crystals do not form a rigid connected network affecting the shape of the growing bubbles nor do their local concentrations appear to vary away from the bubble walls (e.g. Fig. 1f). The absence of bubble deformation coupled to the less localised and polydisperse bubble size distribution enhance the degree of packing of the bubbles and therefore leads to an increase of the percolation threshold (Blower 2001) compared with the brittle-viscous regime. Hereafter, we will show that the crystals also influence the percolation threshold, connectivity and permeability in this viscous regime, but that this effect is lower compared with the brittle-viscous regime.

### Comparison with previous vesiculation experiments involving crystal-rich magmas

**Fig. 8:**
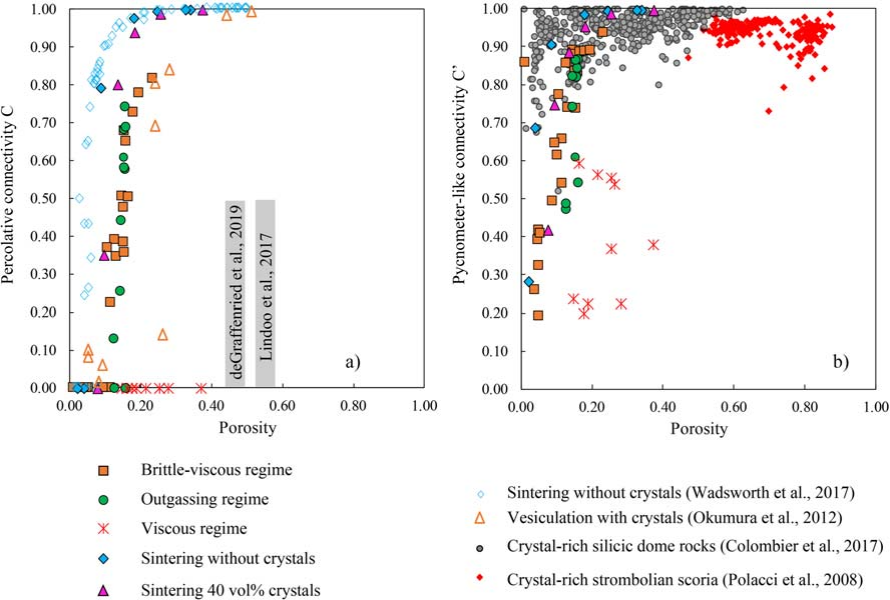
Connectivity-porosity relationships in experimental products and natural crystal-rich volcanic rocks. **a** Evolution of percolative connectivity *C* as a function of porosity during vesiculation in the *brittle-viscous* regime (orange squares), *viscous* regime (red crosses), *outgassing* regime (green circles) and *sintering* regime without crystals (blue solid diamonds) and with 40 vol.% quartz crystals (pink triangles). The experimental data are compared with literature data for previous vesiculation experiments of crystal-rich magmas (orange open triangle from Okumura et al. 2012; grey fields indicating the location of *Φ*_*C*_ from Lindoo et al. 2017 and deGraffenried et al. 2019) and in situ sintering experiments (blue open diamonds; Wadsworth et al. 2017). A percolative definition of connectivity was used in this plot. **b** comparison of *C*′–*Φ* trends from vesiculation and sintering experiments to a wide range of crystal-rich andesitic, trachytic and dacitic dome rocks (grey circles) and crystal-rich Strombolian scoria (red diamonds) compiled in Colombier et al. (2017). A pycnometer-like definition of connectivity *C*′ was here measured from the tomography images in order to compare with the natural data

We compiled values for *Φ*_*C*_ and *C* – *Φ* trends from the literature on vesiculation experiments on crystal-bearing magmas and compare them with our data in Fig. 8a. We find that decompression experiments using low viscosity and moderately crystalline basaltic melts (*ϕ*_*x*_~ 0.15–0.35) yielded high values up to *Φ*_*C*_ ~ 0.56, consistent with our experiments in the viscous regime (Lindoo et al. 2017). Decompression experiments using rhyolitic obsidian powder seeded by corundum crystals with similar synthesis conditions yielded different results (Okumura et al. 2012; deGraffenried et al. 2019). Okumura et al. (2012) found *C* – *Φ* trends resembling our data in the brittle-viscous regime suggesting a percolation threshold *Φ*_*C*_ < 0.3 with starting bulk crystallinities of *Φ*_*x*_=30–50 vol.%. In the same study, all experimental samples had permeabilities below the detection limit (10^−15^ m^2^), even at high bulk porosities (*Φ* = 0.51) suggesting that *Φ*_*C*_ > 0.51. This is consistent with a more recent study by deGraffenried et al. (2019) that used similar starting material and that obtained percolation thresholds (*Φ*_*C*_ = 0.45–0.50) for initial crystallinities of *Φ*_*x*_ = 20–40 vol.%. deGraffenried et al. (2019) also used permeability measurements with similar detection limits to constrain *Φ*_*C*_. The fact that connectivity and permeability data suggest different percolation thresholds in Okumura et al. (2012) might be explained by samples that reached percolation but that maintained permeabilities lower than 10^−15^ m^2^. We therefore propose a range of percolation thresholds of *Φ*_*C*_ = 0.30–0.51 for these decompression experiments on rhyolitic magmas seeded by corundum crystals (Okumura et al. 2012; deGraffenried et al. 2019). Additional differences in the percolative behaviour of these initially similar systems might be related to distinct initial water content, pressures and temperature used during decompression in these two studies.

Other studies found significantly lower percolation thresholds combining experimental, numerical and theoretical studies on crystal-rich magma analogues (Huber et al. 2012; Oppenheimer et al. 2015; Parmigiani et al. 2016). Oppenheimer et al. (2015) conducted Hele-Shaw cell experiments on three phase, magma analogues with solid particle volume fractions of 20 to 54 vol.% and found low percolation thresholds due to enhanced bubble migration via finger- and fracture-like pore geometries. Huber et al. (2012) and Parmigiani et al. (2016) also found that crystals enhance percolation and outgassing via bubble migration via fingering pathways. These studies suggest that the formation of stable finger- and fracture-like pore structures allow percolation at a range of *Φ*_*C*_ = 0.05–0.20, similar to the low percolation thresholds and pore geometries observed here in the brittle-viscous regime. Although this compilation of experimental data suggests a broad range of percolation thresholds for vesiculation of crystal-bearing magmas, most of these studies suggest that the presence of crystals leads to a reduction of *Φ*_*C*_ compared with crystal-free systems vesiculated at similar conditions (Lindoo et al. 2017; deGraffenried et al. 2019).

### Comparison of natural and experimental bubble geometries

Our experiments capture the same geometric evolution of pore spaces as found in crystal-rich natural volcanic rocks. Fracture-like chains of coalesced vesicles acting as permeable pathways can be observed for instance in trachytic and andesitic crystal-rich volcanic rocks (Fig. DR4 in the Electronic supplementary material). Formation of these bubble chains is likely enhanced locally in areas of high crystallinity. After complete coalescence and increase of connectivity, individual bubbles in these chains cannot be further distinguished and instead form permeable pore pathways (Fig. 3c). In the transition from outgassing to viscous regimes and with associated reduction of viscosity, these bubble chains form smooth pathways (Fig. 3d) that resemble large interconnected vesicles found in basaltic Strombolian scoria which coexist with spherical, small individual vesicles (e.g. Polacci et al. 2008, Fig. 1c).

Such textures in silicic and basaltic crystal-rich volcanic rocks are therefore indicative of percolation, outgassing and pore collapse in melts of different viscosities. All these observations confirm that crystals promote initial permeable gas escape and effusive activity in crystal-rich magmas.

### Implications for outgassing and eruptive style of crystal-rich magmas

In order to verify that our vesiculation and sintering experiments are relevant to infer natural volcanic processes occurring during volcanic eruptions involving crystal-rich magmas, we compare our connectivity data with literature data compiled for crystal-rich volcanic rocks. As our experiments involved no decompression, we restrict our comparison to shallow magmatic bodies such as lava domes, plugs or degassed caps in which vesiculation induced by heating may be significant and influence effusive-explosive transitions (Lavallée et al. 2015).

In Fig. 8b, we plot the *C*′ vs. *Φ* trends for experimental and natural crystal-rich silicic (andesitic, trachytic and dacitic) dome rocks and crystal-rich basaltic scoria from Stromboli volcano. We observe that the natural crystal-rich silicic dome rocks, often associated to Vulcanian activity, are explained by the low percolation thresholds observed during vesiculation in the brittle-viscous regime at high viscosity (> 10^6^ Pa s) and high overpressure (1–4 MPa) and/or during the sintering experiments (Fig. 8b). We show that local brittleness can connect pores without fragmenting the material entirely. In turn, this suggests that mixed viscous-brittle behaviour may play a role in decreasing the percolation threshold and on the switch between open- and closed-system degassing in silicic crystal-bearing magmas.

On the other hand, basaltic scoria from Stromboli show *C*′–*Φ* trends that suggest higher percolation thresholds such as those found in the *viscous* regime (*Φ*_*C*_ > 0.37) with low viscosity (< 10^6^ Pa s), low overpressure (0.1 MPa) crystal-rich magma (Fig. 8b). Strombolian activity is commonly characterised by magmas with low melt viscosities and low gas overpressure, that experience important crystallisation, gas percolation and outgassing close to the magma free surface at the top of the conduit (e.g. Gurioli et al. 2008; Polacci et al. 2008; Belien et al. 2010; Leduc et al. 2015). These natural conditions are nicely reproduced by our experimental conditions in the viscous regime. The main difference is that the percolation threshold is likely reached in the magma before or after fragmentation during Strombolian activity, whereas *Φ*_*C*_ was not reached in the *viscous* regime in our experiments. It should be noted that brittle failure may also occur in nature for crystal-rich basaltic magmas at critical conditions of crystallinity, viscosity, strain rate, discharge rate and accumulation of elastic stress (Namiki and Tanaka 2017; Moitra et al. 2018), although these conditions were not met in our experiments.

**Fig. 9:**
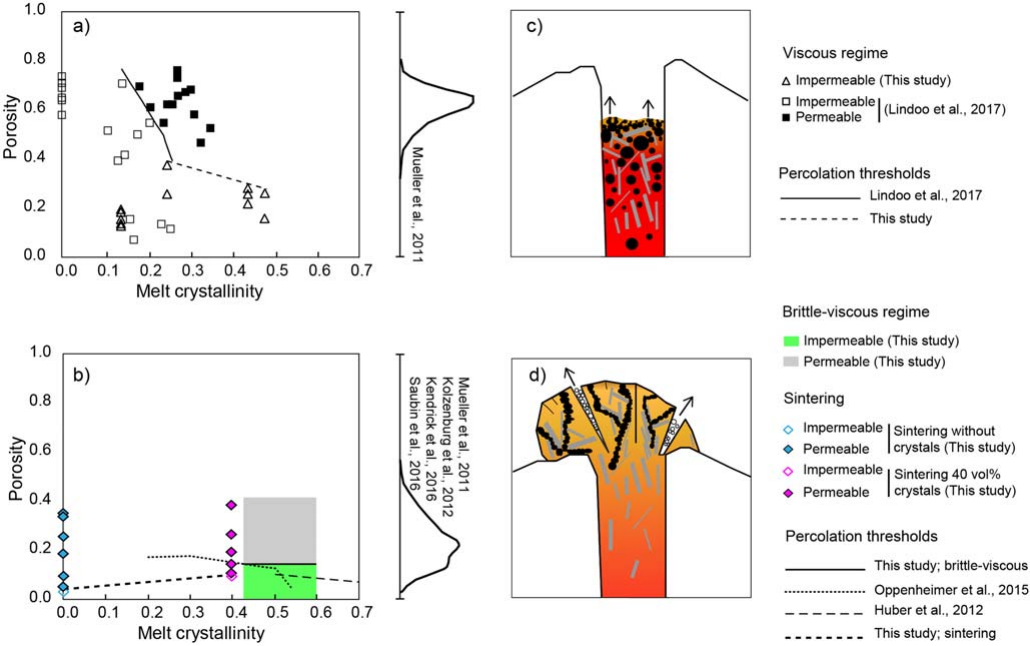
Conceptual model illustrating the influence of crystallinity on the percolation threshold *Φ*_*C*_ in the different regimes. In (**a**) and (**b**), open and solid symbols represent impermeable and permeable data for the *viscous* and *sintering* regimes, respectively. In (**b**), the impermeable and permeable data for the *brittle-viscous* regime are represented by a green and grey area, respectively. **a**) Percolation thresholds found for the viscous regime. The solid curve corresponds to the approximate location of the percolation threshold at low porosity based on results from this study and Lindoo et al. (2017). The dashed curve corresponds to the minimum values of the percolation threshold at high porosity based on experimental results of this study. The distribution of porosity for crystal-rich basaltic scoria from Strombolian activity is represented on the right of the plot (compiled from Mueller et al. 2011b). **b** Low percolation thresholds found in the brittle-viscous and sintering regime and in other studies on crystal-rich magma analogues in which fracture-like bubble coalescence was also observed (Huber et al. 2012; Oppenheimer et al. 2015) for a similar but not identical process of filter-pressing and gas-fingering. Here, our experiments fall at high *ϕ*_*x*_ only (0.4–0.6) because this is the crystallinity local to the bubble walls from which the fracture-like pores grow (termed *ϕ*_*x*_′; see main text). The distribution of porosity data for silicic crystal-rich effusive volcanic rocks is illustrated on the right of the plot (compiled from Mueller et al. 2011b; Kolzenburg et al. 2012; Kendrick et al. 2016; Saubin et al. 2016). The sketches at the right to (**c**) and (**d**) represent a schematic of the volcanic system-type we expect to be relevant. The dashed line in (**b**) for sintering is to guide the eye only

We provide a conceptual model allowing to link the percolation threshold to crystallinity for different conditions of melt viscosities, gas overpressure and pore geometries relevant to specific volcanic scenarios (Fig. 9). We combine our data in the viscous regime with the experimental data of Lindoo et al. (2017) on low viscosity basaltic melts to constrain the evolution of *Φ*_*C*_ with *Φ*_*x*_ (Fig. 9a). The data from Lindoo et al. (2017) suggest an overall reduction of *Φ*_*C*_ with the addition of crystals in this regime, which means that low-viscosity crystal-bearing magmas can result in permeable open-systems at lower *Φ* than crystal-free systems. The percolation threshold was not reached in the viscous regime in our experiments, but the maximum porosity values match with the trend of *Φ*_*C*_ with *Φ*_*x*_ observed by Lindoo et al. (2017).

Our data suggest that this reduction of the percolation threshold in low viscosity, low overpressure, crystal-rich magma may favour outgassing and quiescent, passive degassing or effusive activity occurring at shallow levels of the conduit prior to mild explosive Strombolian activity. We note that a compilation of *Φ* for volcanic rocks produced in Strombolian explosive eruptions straddles the range of permeable experimental products in the viscous regime for all crystallinities (Fig. 9a; Mueller et al. 2011b). On the other hand, the pore geometries, low percolation thresholds and C- *Φ* trends observed in the brittle-viscous*,* outgassing and sintering regimes allow to explain outgassing processes occurring at shallow levels in crystal-rich silicic domes. These low *Φ*_*C*_ are similar to those proposed for bubble migration via finger- and fracture-like bubble chains in crystal-rich magmas (Fig. 9b; Huber et al. 2012; Oppenheimer et al. 2015; Parmigiani et al. 2016). Comparing the range of *Φ* recorded in silicic, crystal-rich dome-rocks worldwide (Mueller et al. 2011b) to our experiments, we find that they straddle the porosities of the permeable samples defined here (Fig. 9b).

We conclude that at similar crystallinity, viscosity and gas overpressure are key parameters controlling the eruptive style of crystal-rich magmas and changes in these parameters may explain transitions between Vulcanian and Strombolian activity. Additional parameters such as crystal wettability, relative size of crystals and bubbles, crystal aspect ratio may also have an influence on bubble nucleation, deformation, distribution and connectivity (Okumura et al. 2012) and should therefore also be considered in future studies.

## Conclusion

We have here quantified the percolation threshold during vesiculation and sintering of crystal-bearing magma analogues with a range of viscosities (10^3^–10^12^ Pa s) and gas overpressures (0.1–4 MPa). This allows us to parameterise our results and compare them with suites of natural volcanic rocks, allowing us to link the percolation threshold in crystal-rich magmas to eruptive style. This allows us to reach the following conclusions:

- When our data is combined with data from previous work (Lindoo et al. 2017), percolation and outgassing in crystal-rich magmas can be explained by variations in crystallinity, melt viscosity and gas overpressure.
- At low viscosity (< 10^6^ Pa s) and overpressure (0.1 MPa), vesiculation progresses in a viscous regime characterised by classic viscous, bubble-growth regime in which spherical bubbles grow and coalesce while pushing the crystals away, developing permeability at relatively high percolation thresholds (*Φ*_*C*_ > 0.37).
- At high viscosity (> 10^6^ Pa s) and high overpressure (1–4 MPa), we observed a brittle-viscous regime in which gas localised on fracture-like expanding pores between crystal domains, resulting in a lower percolation threshold (*Φ*_*C*_ = 0.17) compared with the viscous regime.
- Sintering experiments in which initially fragmental material welds until it becomes impermeable yield low percolation thresholds. The addition of crystals appears to increase the percolation threshold during densification.
- Percolation during vesiculation followed by gas escape and densification can produce a hysteresis in the connectivity-porosity path, consistent with previous studies (Rust and Cashman 2004; Okumura et al. 2013).
- Low percolation thresholds (*Φ*_*C*_ = 0.04–0.17) during vesiculation of crystal-rich, high-viscosity (> 10^6^ Pa s) and high-overpressure (1–4 MPa) magma analogues and during sintering are consistent with *C* – *Φ* relationships of natural crystal-rich silicic domes. We propose that brittle-viscous vesiculation and sintering might therefore promote outgassing and effusive activity during dome-forming eruptions or prior to Vulcanian activity.
- Crystals also lead to a reduction of the percolation threshold during vesiculation in the viscous regime (Lindoo et al. 2017). The percolation thresholds, low viscosity (< 10^6^ Pa s) and low overpressure (0.1 MPa) in this regime are suitable for comparison with crystal-rich scoria that experienced crystallisation and outgassing prior to Strombolian eruptions.

The percolation threshold is a key parameter in the switch between open-system (promoting gas escape) and closed-system (promoting gas overpressure) degassing in erupting magma. As shown here, this percolation threshold may be reached cyclically via vesiculation and densification in shallow crystal-rich magmas, which may explain the frequent shifts between effusive and explosive phases at active volcanoes involving crystal-rich magmas (deGraffenried et al. 2019). Increasing our understanding of the percolation threshold and its controlling parameters in crystal-rich and crystal-free magmas will be a vital step to refine numerical models of degassing and fragmentation and hazard assessment related to effusive-explosive transitions.

## Electronic supplementary material

ESM 1 (DOCX 1926 kb)
