# Supplementary material for: In situ observation of the percolation threshold in multiphase magma analogues
Source: Bull Volcanol. 2020 Mar 4;82(4):32. doi: 10.1007/s00445-020-1370-1 (PMC7056709; doi:10.1007/s00445-020-1370-1)
Supplement: Supplementary file 1 — (DOCX 1926 kb) [file 445_2020_1370_MOESM1_ESM.docx]

**DATA REPOSITORY FOR**

*In situ* observation of the percolation threshold in multiphase magma ANALOGUES

**Colombier, M.^1*^, Wadsworth, F.B.^2^, Scheu, B.^1^, Vasseur, J.^1^, Dobson, K.J.^2^, Caceres, F.^1^,
Allabar, A.^3^, Marone, F.^4^, Schlepütz, C.M.^4^, Dingwell, D.B.^1^**

*^1^Earth and Environmental Sciences, Ludwig-Maximilians-Universität, Theresienstr. 41, 80333 Munich, Germany. ^2^Department of Earth Sciences, Durham University, Science labs, Durham DH1 3LE, United Kingdom. ^3^University of Tubingen, Germany. ^4^Swiss Light Source, Paul Scherer Institute, Villigen, Switzerland.*

This data repository contains 3 principal items:

1. Supplementary methodology description and error analysis (including Figure DR2 and Table DR1)
2. A quantitative comparison of connectivity definitions pertinent to the analysis in the main text (including Figure DR3)
3. Textural evidence for the formation of fracture-like bubble chains in natural crystal-rich silicic volcanic rocks
4. Supplementary methodology
   1. *Temperature calibration*

As the TOMCAT laser heating system only provides intensity values during the experiments, we had to calibrate the experimental temperatures. We performed a sintering experiment on a granular pack of soda lime spherical glass beads (63-90 µm in diameter). The evolution of inter-granular porosity during sintering allowed us to estimate the evolution of viscosity of the melt, which could then be converted to a temperature (Wadsworth et al., 2014). Figure DR1 shows the linear relationship found between intensity and temperature for intensities ranging from 15 to 21A. This linear relationship was then extrapolated at higher temperatures. This same methodology has been applied elsewhere for calibration of the same laser heating system (Wadsworth et al., 2017).

We derived the uncertainty on the temperature recording and the heating rate applied by performing one constrained glass beads sintering experiment in which all the parameters pertaining to describing the kinetics are known. We used the sintering model (e.g. Wadsworth et al., 2017) on the resultant dataset to fit for the dwell temperature and the heating rate, thereby retrieving the standard error on the fit parameters form the least-squares minimization.

- 1. *Textural analysis*

During synthesis of the magma analogues by sintering of glass beads and quartz crystals, several additional crystals grew leading to an increase of the crystallinity. Most of these additional crystalline phases are devitrite crystals that are a typical crystalline phase formed during sintering of soda lime glass beads (e.g., Knowles and Thompson, 2014). These commonly occur in clusters of needle-like crystals with a wide range of aspect ratios (Fig. 1 in the main text). The evolution of the textural properties such as crystallinity, porosity, connectivity and permeability of the magma analogues during the vesiculation and sintering experiments are given with the experimental conditions in Table DR1. The crystallinity at the same time step as the connectivity are what are given in all relevant figures.

- 1. *Scanning procedure, image processing and error estimation*

60 to 300 scans were taken for each experiment. Each 3D scan (1001 projections per scan over 180 degree rotation, pixel resolution of 1.6 µm) was acquired in 1 second (1001 projections) during constant rotation at 0.5Hz. A 5 second wait time between scans ensured all 3D images were spatially registered and gave suitable temporal resolution for all observed changes in pore structure. The projection data were reconstructed into 3D volumes at TOMCAT using the standard pipeline (Marone et al., 2017) (direct Fourier method, ring and zinger suppression, no phase retrieval as contrast enhancement would be detrimental to processing pore quantification and would still be unable to resolve the crystal phases).

The 3D images were processed using Avizo™ software. A volume of interest (VOI) of approximately $1.26\times{10}^{8}$ voxels was cropped in all scans except in two scans r43s2_80 and r38s2_150 for which a much higher VOI was chosen (VOI > $5.53\times{10}^{8}$voxels) due to the presence of large bubbles in the bubbly regime. We chose a large enough VOI to ensure representativeness and reproducibility of the results. We kept the VOI at the same location throughout the scans, thus supressing the effect of heterogeneities in the magma analogues. The VOI was taken at the same height in the sample as the height at which the VOI for the T-calibration sintering run was taken, in order to account for T-gradients within the sample. Segmentation of the greyscale images was performed using auto thresholding (AT) and top hat (TH) modules. Two AT modules were tested with the following parameters: *Auto threshold* low, *Interpretation* 3D, *Mode* other, *Criterion* moments and two different *Input ranges* 90-170 and 90-190, respectively. The former AT with *Input range* 90-170 yielded more realistic segmentation results for porosity and pore connectivity than the latter, but did not include the small isolated vesicles in the analysis (e.g. Fig. DR2). Both methods however led to an overestimation of pore connectivity because it also counted artificial bubble walls and rims as connected porosity (Fig. DR2 e). We therefore applied the top hat module on the segmented images obtained with AT 90-190 in order to provide more realistic porosity and connectivity values and to include the small vesicle population. Top hat consisted in five steps of segmentation: (i) first three steps of *Type* white, *Kernel shape* Ball, *Precision* precise with successive *sizes* of 11, 5, 3 with corresponding lower *Masking* bound of 8, 6 and 5, respectively (removing the artificial walls); (ii) two steps of *Type* black, *Kernel shape* Ball, *Precision* Precise and *sizes* of 5 and 3 with corresponding lower *Masking* bounds of 22 and 13 (adding small isolated populations back to porosity). This step allowed to remove artificial walls and rims around the bubbles and to take into account small bubbles that were not previously segmented (e.g., Fig. DR2 f).

After segmentation we measured the porosity by counting the number of voxels corresponding to pores and dividing them by the VOI. We then measured the connected porosity by counting the number of connected voxels in the three directions using *axis connectivity* module and considering two neighbouring vesicles as connected if they shared at least one voxel face. The connectivity C was then defined as the ratio between the connected and the total porosity.

We compared porosity and connectivity values obtained using the most realistic auto thresholding (AT 90-170) and the most overestimated auto thresholding followed by top hat segmentation on three datasets. The porosity analysis yielded systematically similar results (Fig. DR2) but different values of C for C>0. As the values of C are clearly more realistic for the combined segmentation using Auto thresholding and top hat, we used this strategy to treat all the scans and propose that the error on porosity and connectivity with this technique are negligible (with a maximum error of 0.1 for C). We also note that the percolation threshold is not affected by the choice of segmentation as values of C below Φ_c_ are zero with the two techniques. We also compare our chosen segmentation result with the most unrealistic AT method (AT 90-190), and show that the overall trend of Φ-C is similar (Fig. DR3).

During vesiculation, the onset of connectivity occurs at the percolation threshold via the formation of bubble chain. Individual bubbles are still visible at this stage (e.g., Fig. 3b in the main text) and the presence of films smaller than the pixel size (1.6 µm) separating these bubbles could not be resolved. This might cause an overestimation of the connectivity and underestimation of the percolation threshold. When the connectivity is maximum, bubble coalescence is such that the bubble chains clearly form permeable networks and films separating bubbles are absent (e.g., Fig. 3c in the main text). The percolation threshold in the *brittle-viscous* regime was therefore taken as the average porosity between onset (Φ=0.11) and maximum (Φ=0.23) connectivity (see Table DR1), yielding $\Phi_{C1}\sim0.17\pm0.06$.

Pre- and post-experimental sample changes were also analysed by back-scattered SEM (HITACHI SU 5000 Schottky FE) to measure groundmass crystallinities accurately, confirming the synthesis conditions.

1. A quantitative comparison of connectivity definitions

Tomography allows to measure connectivity directed in one direction of the sample, which is more relevant for comparison with permeability. Most of connectivity data available in literature were obtained by pycnometry that allow to measure the connectivity C’ to the exterior of the sample. In our datasets we were able to measure the two types of definitions and compare them (Fig. DR4).

We find that most of the data fit in a linear relationship (Fig. DR4). At complete connectivity, the two methods converge toward the same value of C=C’=1. Instead, even if no percolating vesicle cluster exist and C=0, some vesicles are still connected to the exterior, yielding C’>0. Using this approach, we are now able to provide a correction to the connectivity data measured by pycnometry on natural volcanic rocks and to retrieve a “percolating” connectivity C for these measurements. However, we stress that this method should be tested on samples with a range of size and pore characteristics to allow for an accurate correction of pycnometry data specific to a rock type. The connectivity values for both definitions and for each scan are given in Table DR1.

1. Fracture-like bubble chains in natural crystal-rich silicic rocks

In figure DR5, we provide textural images, obtained on a Cameca SX-100 electron-microprobe at Ludwig Maximilians University, Germany, of a trachytic clast from a Vulcanian eruption at Kilian volcano, France (Colombier et al. 2017). We also provide a BSE image of a crystal-rich clast from Tongariro volcano, New Zealand (Heinrich, personal communication). These images reveal the presence of a fracture-like bubble chains in crystal-rich samples.

**
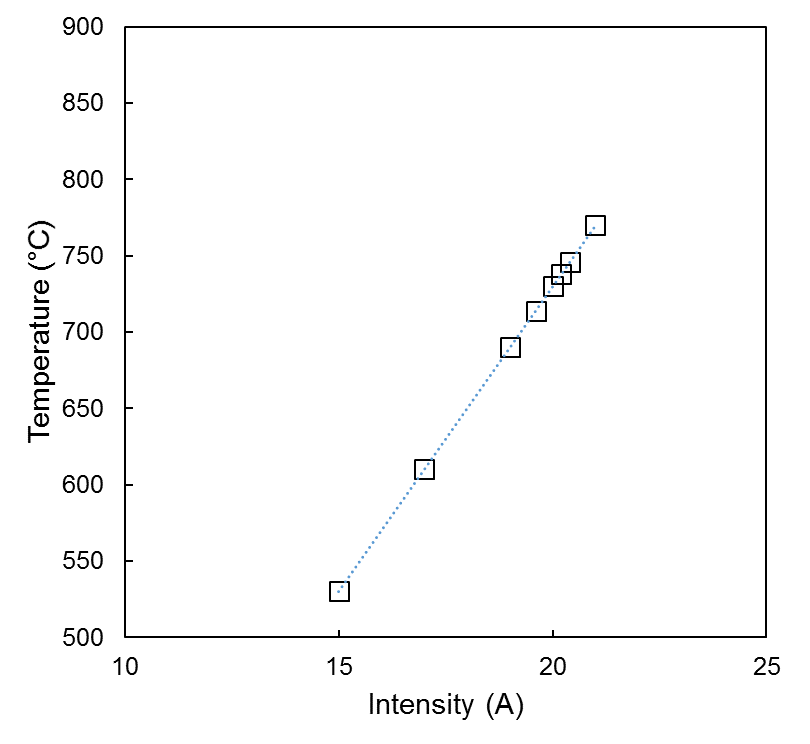
**

**Figure DR1.**

Relationship found between intensity I and temperature T during the temperature calibration using a sintering experiment with intensities ranging from 15 to 21A. The expression for the regression line is as follows: T=40I-70 with a coefficient of determination of R²=0.99.

**Gray scale image AT-90-170 without Top Hat AT-90-190 with Top Hat**


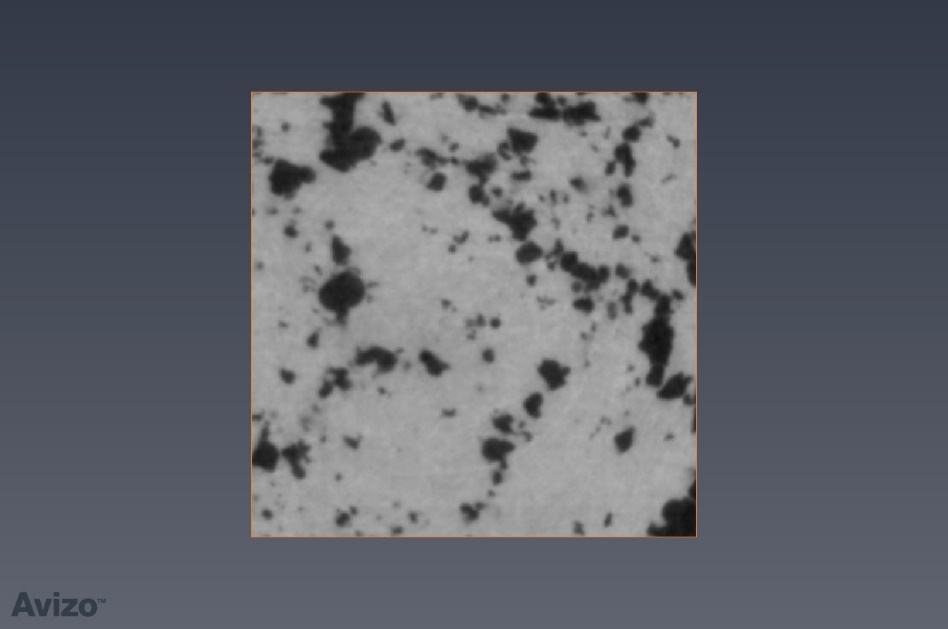

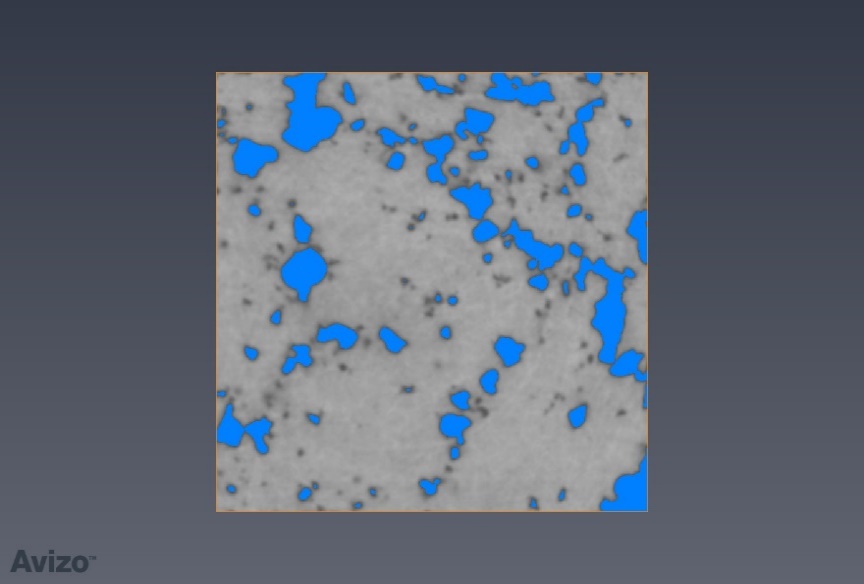

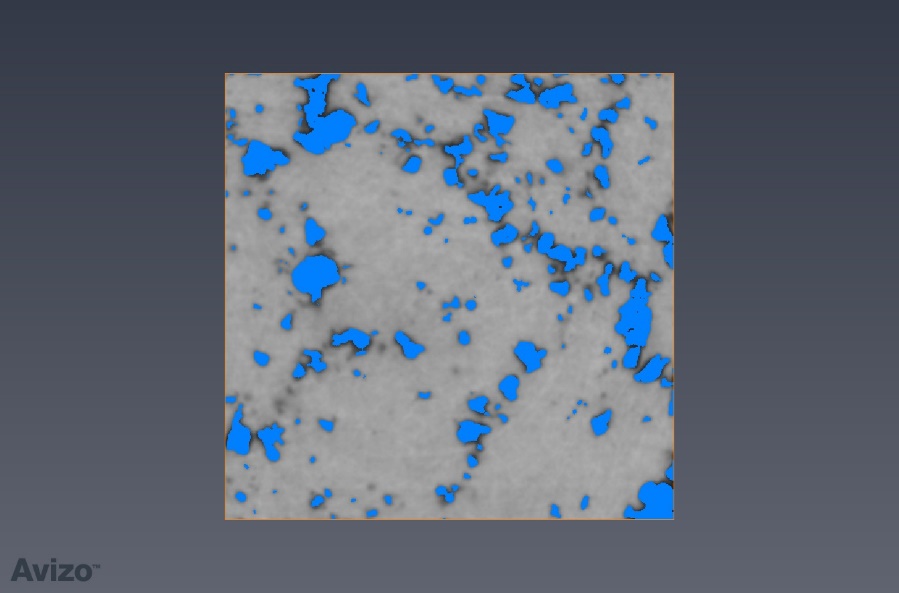


c

b

a


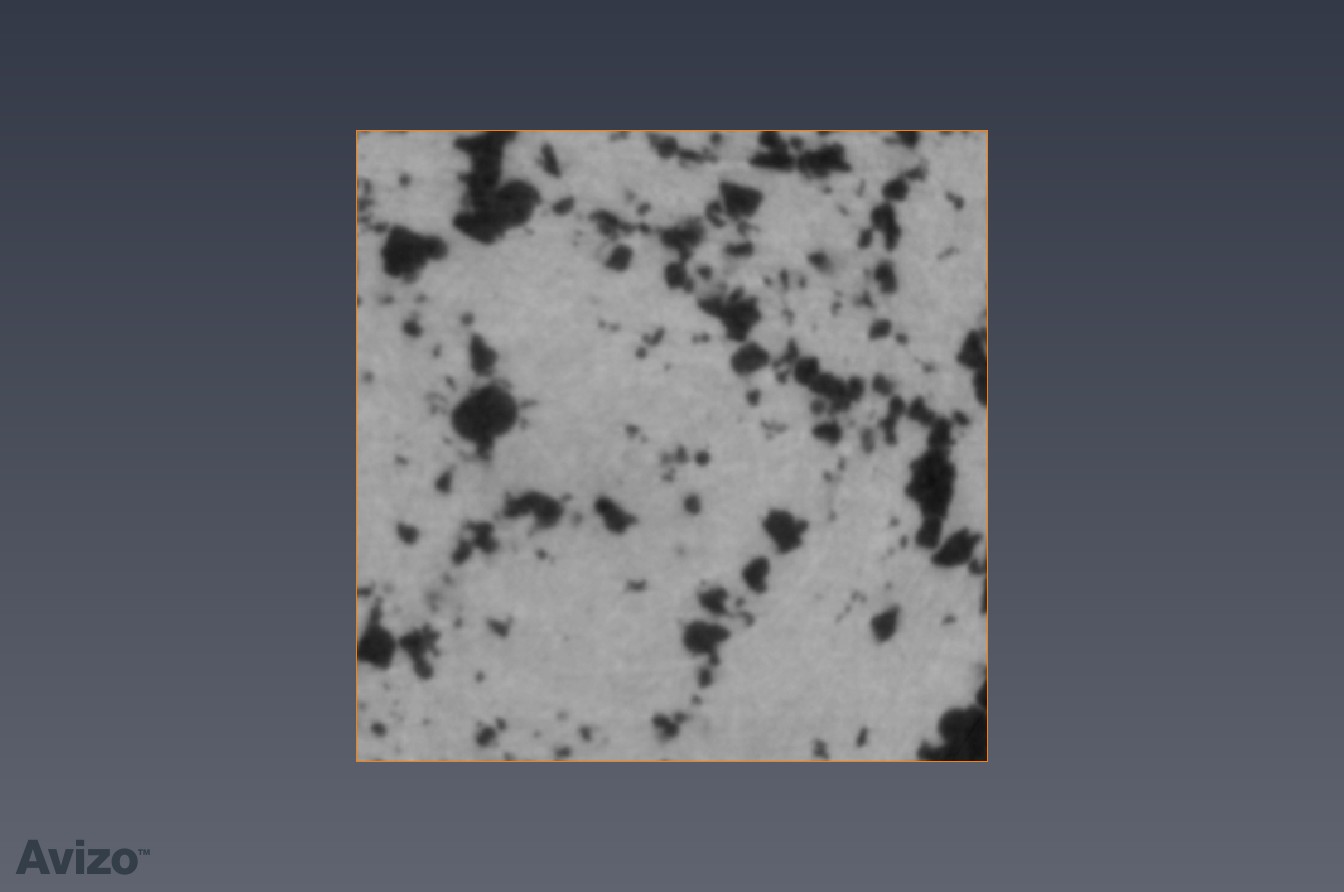

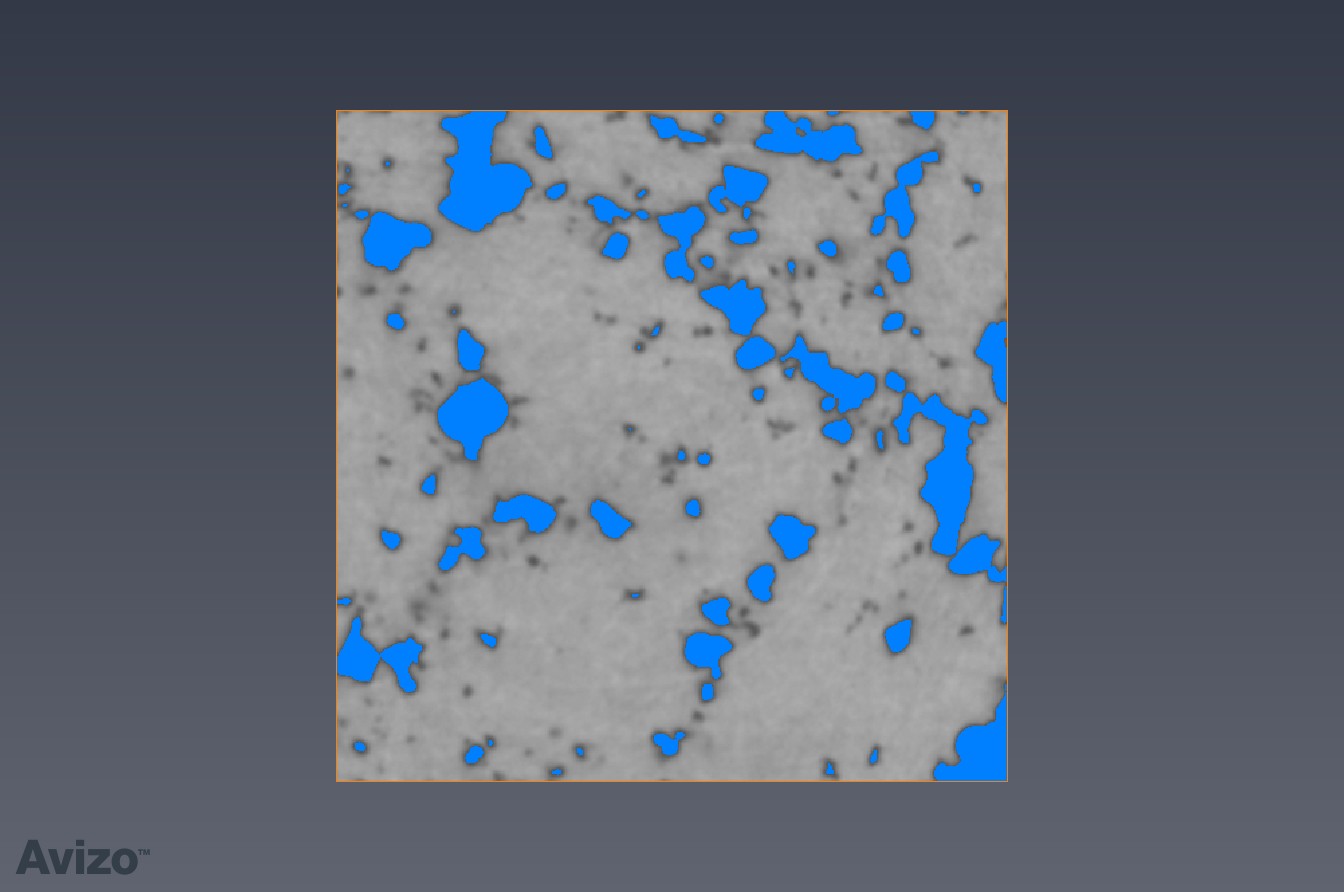

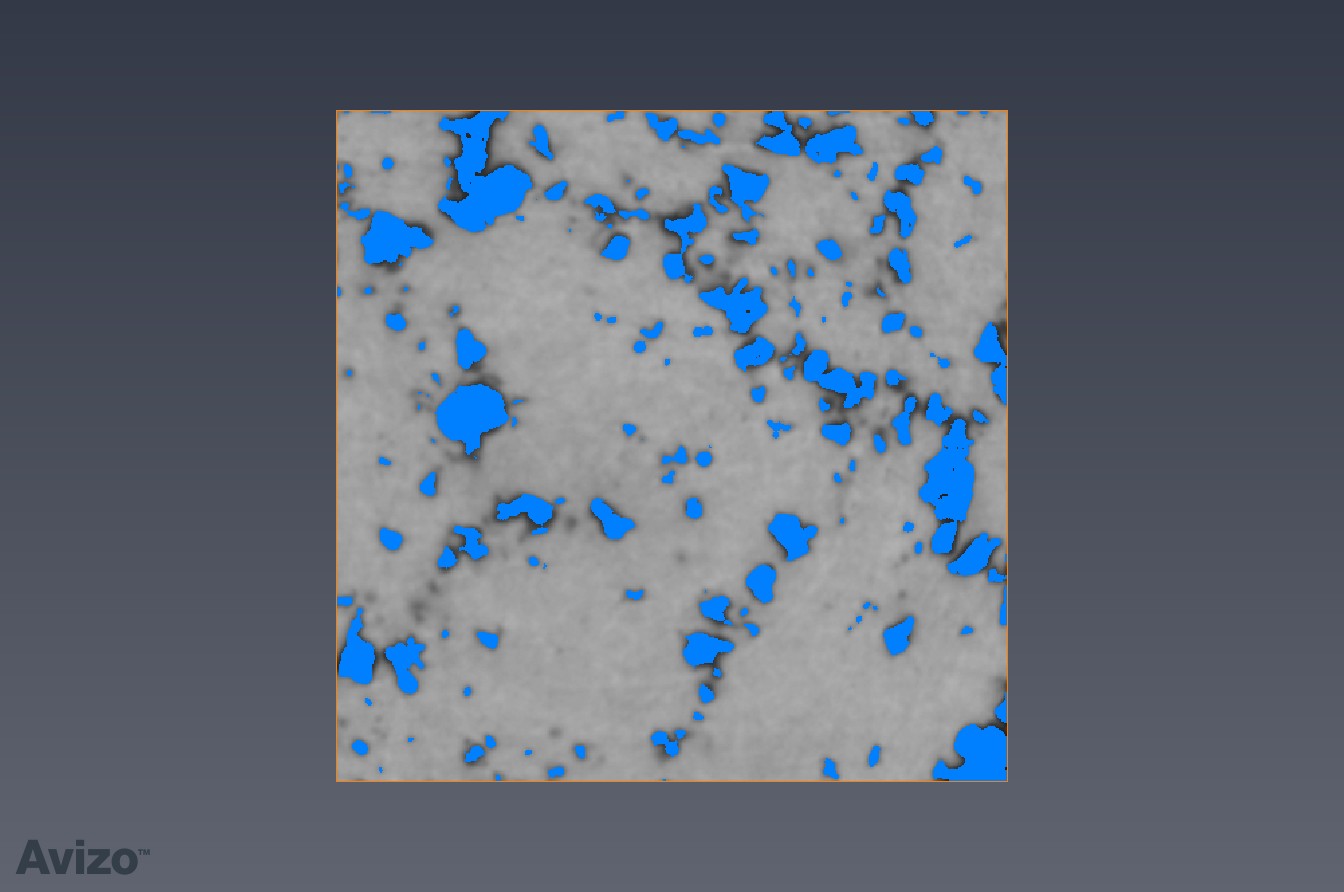


f

e

d

**Figure DR2.** Image processing and error estimation procedure including values of porosity and connectivity for three datasets with the most realistic auto thresholding (AT 90-170) and overestimated auto thresholding followed by top hat (AT 90-190 + TH). a-c: Grayscale and binary images obtained after segmentation. d-f: Zooms of the grayscale and binary images showing the effect of segmentation on pore connectivity. Auto-thresholding without top hat overestimates bubble connectivity by including bubble films in the connected porosity and by excluding small isolated bubbles (e).


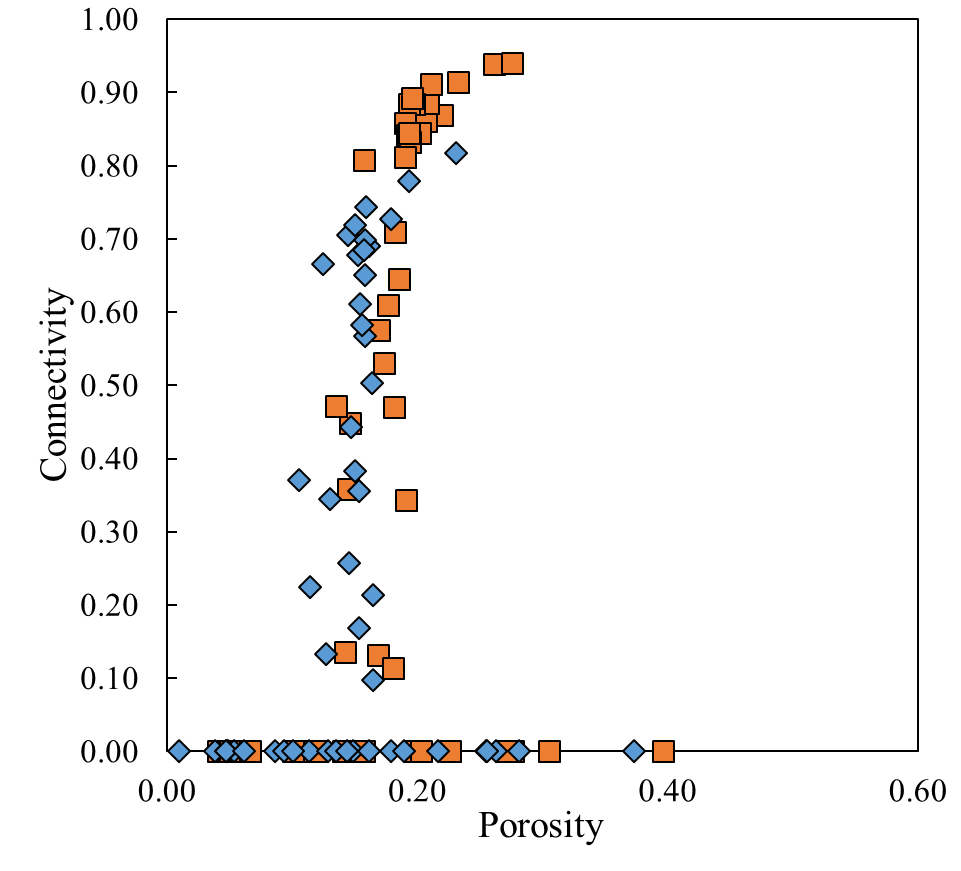


**Figure DR3.**

Comparison between the connectivity-porosity relationships using (i) Auto-thresholding segmentation (AT 90-190) with overestimated porosity and connectivity (orange squares) and (ii) Auto-thresholding (AT 90-190) followed by additional Top hat analysis (blue diamonds) leading to more realistic values of porosity and connectivity. We note that the overall trend is similar with both segmentation techniques.


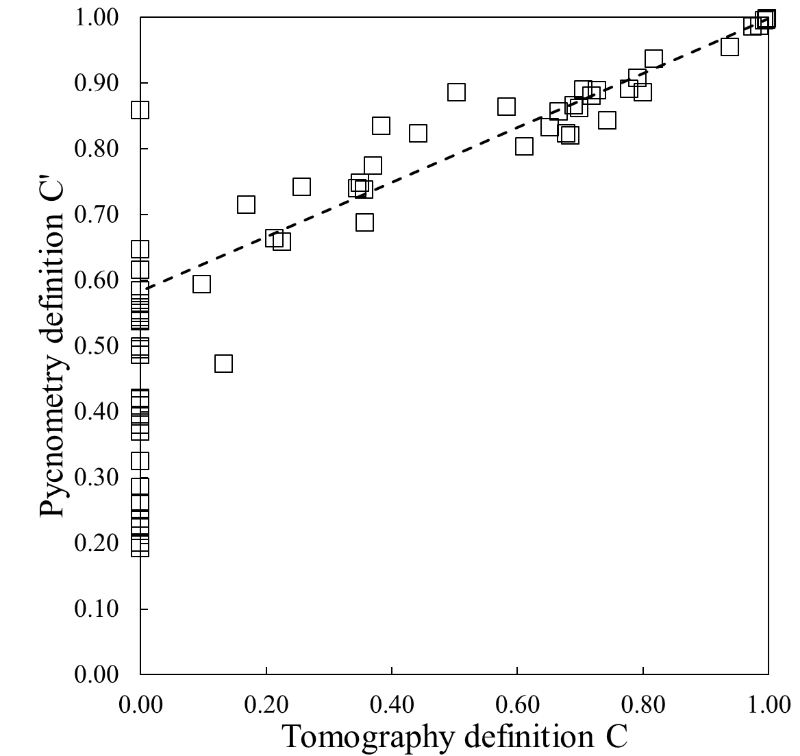


**Figure DR4.**

Comparison of the connectivity values measured in the magma analogues using two different definitions by X-ray micro-tomography. The connectivity C’ was derived using a pycnometry definition in which each vesicle connected to the surface is treated as connected. The connectivity C represents a measure of the fraction of the pores connected from one side of the sample to the opposite. The linear fit between the two definitions for non zero values of C and C’ allow converting the connectivity data on andesitic and dacitic effusive rocks derived by He pycnometry C’ (Colombier et al., 2017a) to percolating connectivities C.


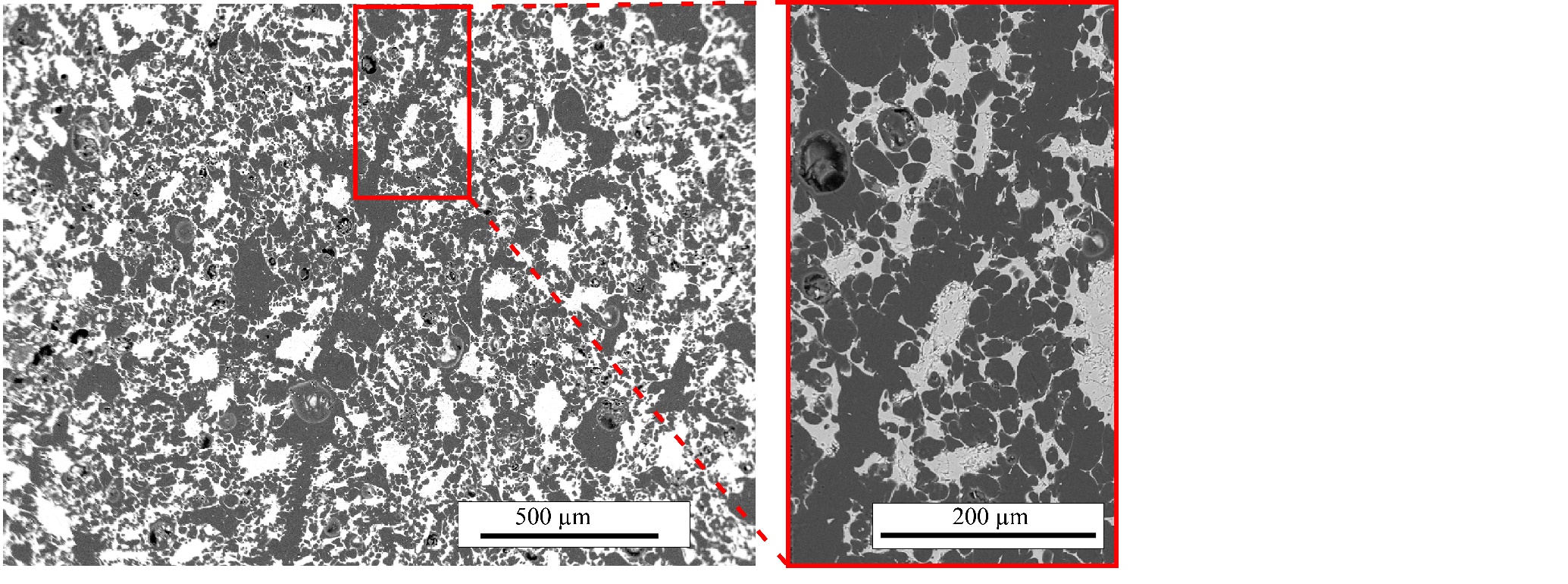


b)

a)


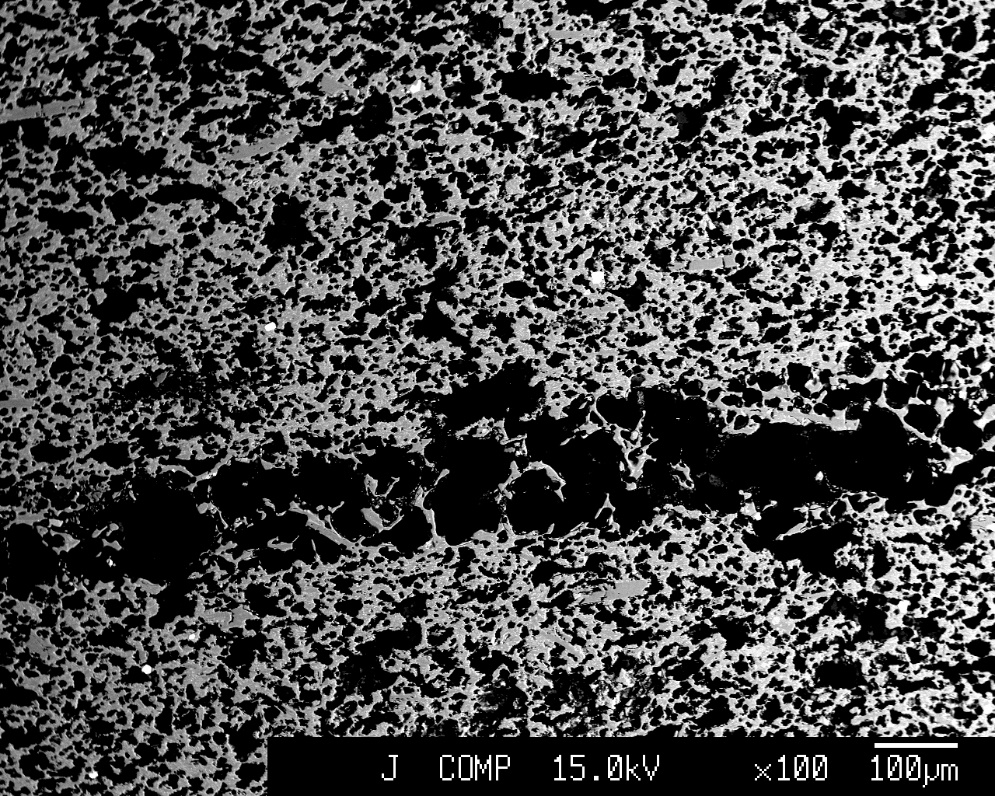


200 µm

**Figure DR5.**

SEM images of system-spanning fracture-like vesicle chains in crystal-rich silicic volcanic rocks. a) Vesicle chain in a trachytic, crystal-rich clast of the 9400 BP Vulcanian eruption of Kilian volcano, Chaîne des Puys, France (Colombier et al. 2017b). The right panel shows a close-up on this fracture-like connecting pathway and clearly indicates that it is formed of a chain of individual coalesced vesicles. b) Vesicle chain in an andesitic crystal-rich clast of Tongariro volcano, New Zealand (Heinrich, personal communication).

**Table DR1**

Experimental conditions and raw data for the magma analogues analysed in this study. Values for bulk crystallinity (Φx), heating rate (HR), time (t), intensity (I), temperature (T), melt viscosity (η), pressure (P), porosity (Φ), percolative connectivity in three directions (C) and pycnometry-like connectivity (C’) are provided. Estimated errors for temperature, viscosity and pressure are also given.

**References**

Colombier M, Gurioli L, Druitt TH, et al (2017) Textural evolution of magma during the 9.4-ka trachytic explosive eruption at Kilian Volcano, Chaîne des Puys, France. Bull Volcanol 79:. doi: 10.1007/s00445-017-1099-7

Knowles, K.M., and Thompson, R.P., 2014, Growth of devitrite, Na_2_Ca_3_Si_6_O_16_, in soda-lime-silica glass: Journal of the American Ceramic Society, v. 97, p. 1425–1433, doi: 10.1111/jace.12922.

F. Marone, A. Studer, H. Billich, L. Sala & M. Stampanoni. Towards on-the-fly data post-processing for real-time tomographic imaging at TOMCAT. Advanced Structural and Chemical Imaging 3, 1, 2017.

Wadsworth, F.B., Vasseur, J., Aulock, F.W. Von, Hess, K.-U., Scheu, B., Lavallée, Y., and Dingwell, D.B., 2014, Nonisothermal viscous sintering of volcanic ash: Journal of Geophysical Research: Solid Earth, v. 119, p. 8792–8804, doi: 10.1002/2014JB011453.

Wadsworth, F.B., Vasseur, J., Llewellin, E.W., Dobson, K.J., Colombier, M., Von Aulock, F.W., Fife, J.L., Wiesmaier, S., Hess, K.U., Scheu, B., Lavallée, Y., and Dingwell, D.B., 2017, Topological inversions in coalescing granular media control fluid-flow regimes: Physical Review E, v. 96, p. 0–6, doi: 10.1103/PhysRevE.96.033113.
